# Impact of obesity on all-cause and cause-specific mortality among critically ill men and women: a cohort study on the eICU database

**DOI:** 10.3389/fnut.2023.1143404

**Published:** 2023-04-21

**Authors:** Shan Li, Wei Zhang, Zhiqing Fu, Hongbin Liu

**Affiliations:** ^1^Department of Cardiology, The Second Medical Center, Chinese People’s Liberation Army General Hospital, Beijing, China; ^2^National Clinical Research Center for Geriatric Disease, Beijing, China; ^3^Department of Outpatient, The Second Medical Center, Chinese People’s Liberation Army General Hospital, Beijing, China

**Keywords:** obesity, sex difference, all-cause mortality, cause-specific mortality, critically ill patients

## Abstract

**Background:**

The effect of obesity on intensive care unit outcomes among critically ill patients and whether there are sex differences have not been well investigated. We sought to determine the association between obesity and 30-day all-cause and cause-specific mortality among critically ill men and women.

**Methods:**

Adult participants who had body mass index (BMI) measurements were included from the eICU database. Participants were divided into six groups according to BMI (kg/m^2^) categories (underweight, <18.5; normal weight, 18.5–24.9; overweight, 25–29.9; class I obesity, 30–34.9; class II obesity, 35–39.9; class III obesity, ≥40). A multivariable adjusted logistic model was conducted with odds ratios (ORs) and 95% confidence intervals (CIs). A cubic spline curve based on the generalized additive model was used to represent the nonlinear association. Stratified analysis and sensitivity analysis were also performed.

**Results:**

A total of 160,940 individuals were included in the analysis. Compared with the class I obesity category, the underweight and normal weight categories had higher all-cause mortality, and the multivariable adjusted ORs were 1.62 (95% CI: 1.48–1.77) and 1.20 (95% CI: 1.13–1.27) for the general population, 1.76 (95% CI: 1.54–2.01) and 1.22 (95% CI: 1.13–1.32) for men, and 1.51 (95% CI: 1.33–1.71) and 1.16 (95% CI: 1.06–1.27) for women, respectively. Accordingly, multivariable adjusted ORs for the class III obesity category were 1.14 (95% CI: 1.05–1.24) for the general population, 1.18 (95% CI: 1.05–1.33) for men, and 1.10 (95% CI: 0.98–1.23) for women. With cubic spline curves, the association between BMI and all-cause mortality was U-shaped or reverse J-shaped. Similar findings were observed for cause-specific mortality, with the underweight category associated with a higher risk of mortality. Class III obesity increased the risk of cardiovascular death among men (OR 1.51; 95% CI: 1.23–1.84) and increased the risk of other-cause death among women (OR 1.33; 95% CI: 1.10–1.61).

**Conclusion:**

The obesity paradox appears to be suitable for all-cause and cause-specific mortality among critically ill men and women. However, the protective effect of obesity cannot be extended to severely obese individuals. The association between BMI and cardiovascular mortality was sex-specific and was more pronounced among men than among women.

**Graphical abstract:**
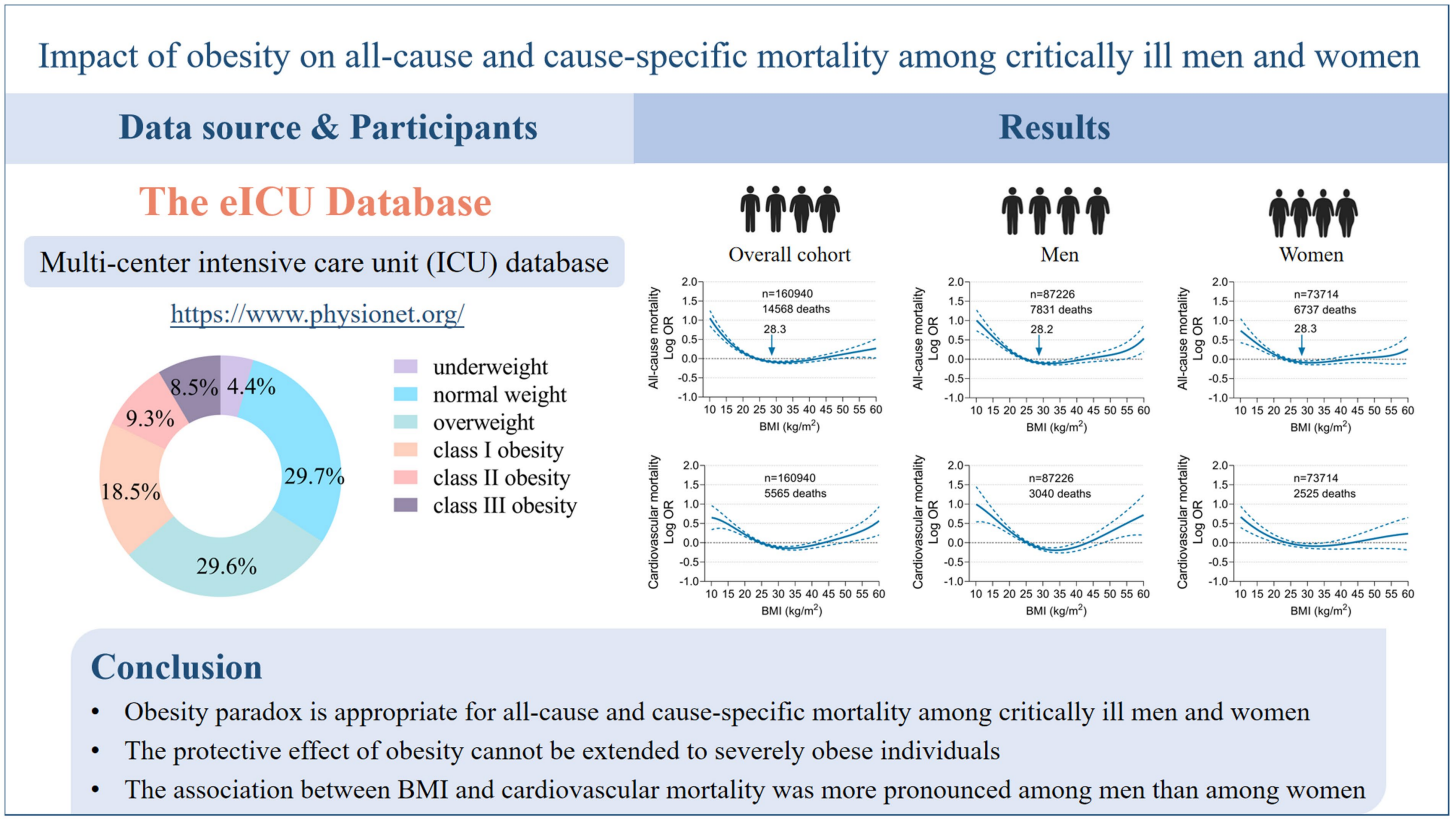

## Introduction

The prevalence and disease burden of obesity is increasing worldwide, posing a substantial public health challenge and clinical concern. By 2025, the global prevalence of obesity is expected to reach 18% for men and 21% for women, and severe obesity will surpass 9% in women and 6% in men (1). Epidemiological studies have revealed that obesity is strongly correlated with a series of pathophysiological disturbances, including atherosclerosis, diabetes, hypertension, chronic obstructive pulmonary disease, renal insufficiency and cancer (2–4). In recent years, the evidence that obesity promotes valvular heart disease, cardiomyopathy and multimorbidity has been further consolidated (5–7). These problems are direct consequences of excessive fat mass or indirect consequences of obesity-related metabolic dysfunction. Due to the high metabolic activity of adipose tissue, abnormal and detrimental adipocyte secretion patterns promote chronic proinflammatory, prothrombotic and procoagulant states. Although obesity carries a range of disease risks, elevated BMI is paradoxically associated with better survival in various clinical settings, including heart failure, atrial fibrillation, nephropathy, sepsis, acute respiratory distress syndrome and critical illness (8–13). This so-called ‘obesity paradox’ phenomenon appears to be more pronounced among men according to several studies (14, 15). However, recent studies on coronavirus disease 2019 (COVID-19) have consistently shown that obesity is independently correlated with severe outcomes and mortality from COVID-19 infection (16, 17). Its pathophysiological mechanisms involve obesity-induced weakened immune response, hypercoagulation and metabolic disorder (18). Therefore, the existence of the obesity paradox in different populations remains controversial.

Critically ill patients admitted to the intensive care unit (ICU) have a variety of systemic diseases, which are more dangerous and have a higher risk of death. Obesity brings greater diagnostic challenges (CT or ultrasound image quality reduction), increased operation difficulty (such as tracheal intubation), and pharmacokinetic and pharmacodynamic changes, which may complicate acute diseases and weaken the effectiveness of evidence-based interventions. Therefore, it is imperative to understand the impact of obesity on the clinical prognosis of these patients. However, reliable data on the relationship between obesity and mortality in critical settings are scarce and discrepant, showing positive, zero, or negative correlations (19, 20). Some studies reported positive results but involved only all-cause mortality and no cause-specific mortality. Moreover, it is now believed that fat mass and distribution vary by sex, and whether there is a sex difference in the association between obesity and mortality is also a matter of concern that has not been well assessed. To address this evidence gap, we analysed data from a large contemporary multicentre ICU cohort to explore whether there is an obesity paradox in all-cause and cause-specific mortality among critically ill patients, and if the obesity paradox exists, the existence of a sex-related difference therein.

## Methods

### Study participants

Data were extracted from the publicly available eICU Collaborative Research Database (eICU-CRD). The eICU-CRD is a telemedicine system developed by Philips Healthcare in cooperation with the Laboratory for Computational Physiology (LCP) of the Massachusetts Institute of Technology to optimize the management of critically ill patients (21). The LCP has previously successfully shared the Medical Information Mart for Intensive Care (MIMIC) database to support academic research and ICU quality improvement (22). The eICU-CRD is a complete and expanded dataset independent of MIMIC, which collects comprehensive clinical data of more than 200,000 ICU encounters from 208 U.S. hospitals. This high-quality data integration system contains a large amount of information on demographic profiles, vital signs, disease severity scores, laboratory parameters, fluid balance, medications, diagnostic codes, treatments, survival status, and hospital-level data, including regional location, teaching status, bed capacity, etc. All data were deidentified, and the requirement for informed consent from patients was waived. Data are free to access after completing the required training course and signing a usage agreement. This study was conducted in accordance with the Declaration of Helsinki. The study is exempt from institutional review board approval due to the retrospective design, lack of direct patient intervention, and the security schema, for which the re-identification risk was certified as meeting safe harbor standards by an independent privacy expert (Privacert, Cambridge, MA) (Health Insurance Portability and Accountability Act Certification no. 1031219-2). One author (Shan Li) obtained database access and was responsible for data extraction (certification number: 46622370). We included individuals admitted to the ICU from 2014 to 2015. The exclusion criteria were (1) age under 18 years, (2) no weight or height data available, and (3) BMI < 10 kg/m^2^ or > 60 kg/m^2^. Finally, 160,940 individuals were included in the analysis.

### Exposure

The primary exposure of interest was BMI, calculated by the formula BMI (kg/m^2^) = weight/height^2^. For this calculation, the weight and height documented at ICU admission were used. According to the international classification criteria, individuals were divided into six categories: underweight, <18.5 kg/m^2^; normal weight, 18.5–24.9 kg/m^2^; overweight, 25–29.9 kg/m^2^; class I or mild obesity, 30–34.9 kg/m^2^; class II or moderate obesity, 35–39.9 kg/m^2^; and class III or severe obesity, ≥40 kg/m^2^.

### Outcomes

The primary outcome was all-cause mortality within 30 days of ICU admission. The secondary outcomes were cardiovascular mortality, infectious mortality, and other-cause mortality. ICU death statistics were determined according to the International Classification of Diseases codes (9th revision). All-cause death was defined as death caused by any cause from the date of admission until the time of death. Cardiovascular death was defined as death from diseases with ICD-9 codes 390–459. Infectious disease death was defined as death from diseases with ICD-9 codes 320–326, 460–488, 566–567, 590, 595, 597, 614–616, 680–686, and 995. Noncardiovascular and noninfectious causes death were defined as other-cause death. Death from acute myocardial infarction was defined as death from disease with ICD-9 code 410, death from heart failure was defined as death from disease with ICD-9 code 428, death from sepsis was defined as death from a condition with ICD-9 code 995, death from ischaemic stroke was defined as death from conditions with ICD-9 codes 430–432, and death from intracranial haemorrhage was defined as death from conditions with ICD-9 codes 433–434.

### Covariates

The following factors were considered for covariate selection: (1) individual-level factors, including age, sex, ethnicity, heart rate, mean blood pressure, disease severity score (Acute Physiology, Age and Chronic Health Evaluation [APACHE] score and Glasgow Coma Scale [GCS] score); (2) clinical risk factors, including primary disease at admission (cardiovascular disease, respiratory disease, digestive disease, genitourinary disease, neurological disease, endocrine disease, trauma, other infectious disease [nonrespiratory, nonurinary, nondigestive tract infections disease]) and prehospital comorbidities (coronary artery disease, stroke/transient ischaemic attack, diabetes mellitus, hypertension, congestive heart failure, peripheral artery disease, chronic obstructive pulmonary disease, renal dysfunction); (3) important treatments, including mechanical ventilation, dialysis and vasoactive drugs; and (4) hospital-level factors, including admission source, geographic location and discharge year.

### Statistical analysis

Statistical analyses were performed using R software (version 3.6.1)^1^ and EmpowerStats (X&Y Solutions, Inc., Boston, MA).^2^ Statistical significance was defined as a 2-sided *p* value <0.05. Continuous variables are presented as the means with standard deviations (SDs) or medians with interquartile ranges (IQRs) and analysed using unpaired t tests or Mann–Whitney U tests depending on their distribution. Categorical variables are presented as numbers with percentages and were compared using the chi-square or Fisher’s exact test. The R package multiple imputation by chained equation (*N*_imputation_ = 5) was used to account for missing data (13.3% for APACHE score, 2.5% for GCS score).

A multivariable logistic regression model was used to examine adjusted ORs for the association between BMI on a categorical scale and all-cause and cause-specific mortality, with the BMI category related to the lowest mortality as a reference. Three models were constructed: Model I unadjusted, Model II adjusted by age, sex and ethnicity, and Model III adjusted by all covariates without selection. The cubic spline curves based on the generalized additive model that adjusted for all covariates were used to visually display the nonlinear relationship between BMI on a continuous scale and all-cause and cause-specific mortality. Stratified analysis was conducted to examine the interaction between BMI and stratified covariates on all-cause mortality by including two or multiple interaction terms with adjustment for predefined covariates. Several sensitivity analyses were conducted to evaluate the robustness of the primary analysis. First, we excluded deaths that occurred within the first 48 h of ICU entry to determine whether the association between BMI and mortality could be explained by a reverse causality of severe disease. Second, we performed a complete case analysis using only the complete data of all covariates to test whether the missing data distorted the current findings. Third, we plotted Kaplan–Meier survival curves by taking the length of ICU stay time as an underlying time scale and censoring at discharge or death to assess whether different statistical methods might change the results.

## Results

### Baseline characteristics

**Figure 1:**
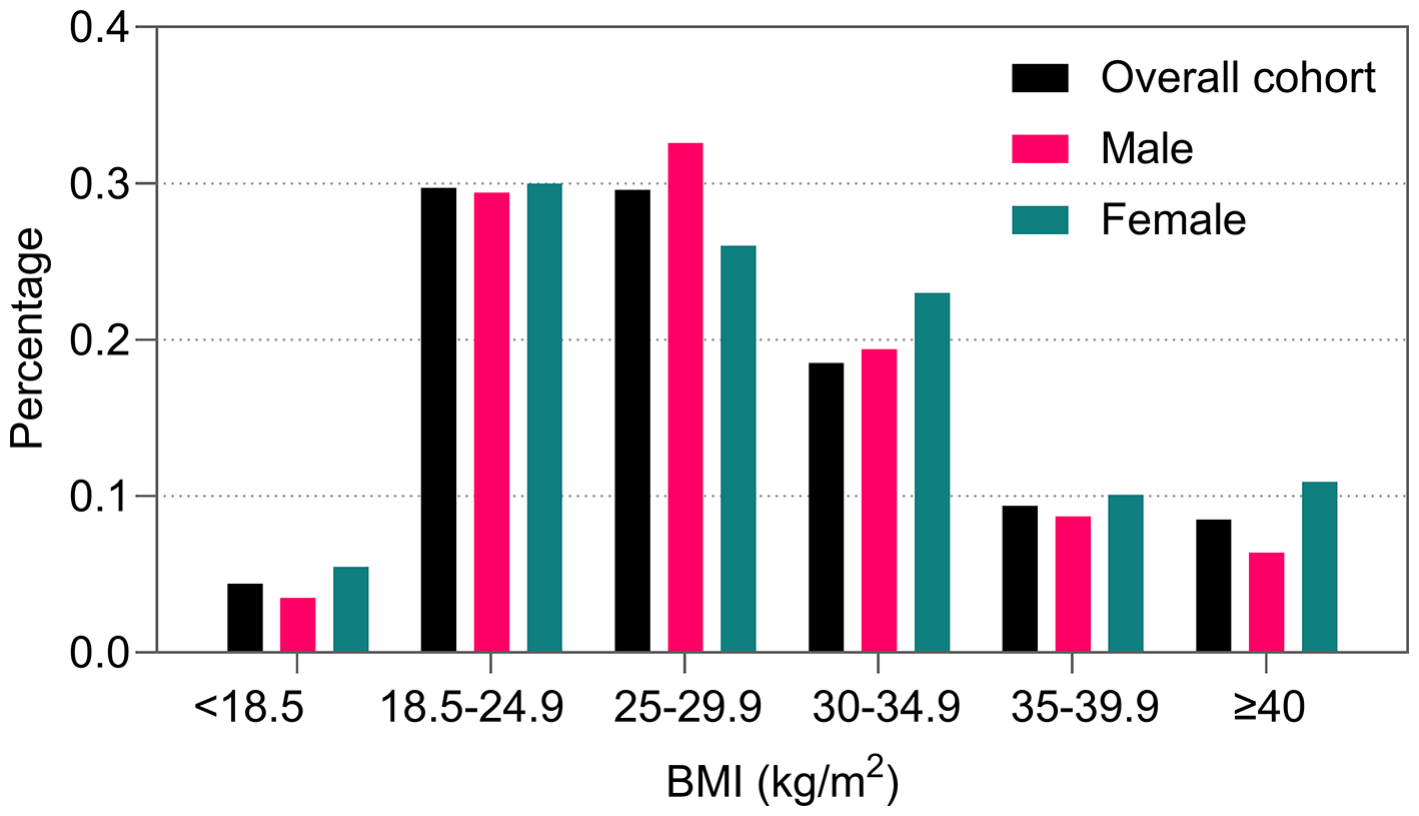
Distribution of BMI categories among men and women.

A total of 160,940 individuals were included in this study (mean [SD] age, 63.2 [17.1] years; 87,226 men [54.2%] and 123,959 Caucasians [77.0%]), 14,568 (9.1%) all-cause deaths, 5,565 (3.5%) cardiovascular deaths, 4,308 (2.7%) infectious disease deaths and 4,695 (2.9%) other-cause deaths were recorded within 30 days of ICU admission. The average BMI was 28.7 (SD 7.6) kg/m^2^, and 58,518 (36.4%) individuals had class I to class III obesity. Figure 1 shows the distribution of BMI categories in the overall population and among men and women. Individuals with a higher BMI were younger and had a higher prevalence of diabetes mellitus, hypertension, chronic heart failure and renal dysfunction. Individuals with a lower BMI were older, had higher APACHE scores, and had a higher prevalence of respiratory disease and digestive disease. More dependence on mechanical ventilation was observed among individuals with class III obesity. Both underweight and class III obese patients had longer ICU stays. Underweight individuals accounted for 4.4% of the total population, resulting in 14.4% of all-cause deaths, which was approximately twice that of class I obese individuals. Baseline characteristics classified by BMI category are shown in Table 1.

**Table 1:** Baseline characteristics of individuals by BMI categories.

BMI, kg/m^2^	<18.5	18.5–24.9	25.0–29.9	30.0–34.9	35.0–39.9	≥40	*p* value
*N* (%)	7,067 (4.4)	47,768 (29.7)	47,587 (29.6)	29,818 (18.5)	15,042 (9.3)	13,658 (8.5)	
Age, years	64.2 ± 19.1	64.1 ± 19.0	64.3 ± 16.7	62.8 ± 15.5	61.1 ± 14.9	58.6 ± 14.2	<0.001
Male, *n* (%)	4,056 (57.4)	22,112 (46.3)	19,170 (40.3)	12,874 (43.2)	7,440 (49.5)	8,062 (59.0)	<0.001
Ethnicity							<0.001
Caucasian, *n* (%)	5,327 (75.4)	36,638 (76.7)	36,710 (77.1)	23,243 (77.9)	11,622 (77.3)	10,419 (76.3)	
African American, *n* (%)	952 (13.5)	4,962 (10.4)	4,724 (9.9)	3,207 (10.8)	1,876 (12.5)	2,058 (15.1)	
Hispanic, *n* (%)	206 (2.9)	1,846 (3.9)	2,001 (4.2)	1,065 (3.6)	492 (3.3)	393 (2.9)	
Asian, *n* (%)	212 (3.0)	1,219 (2.6)	802 (1.7)	299 (1.0)	101 (0.7)	53 (0.4)	
Other/unknown, *n* (%)	370 (5.2)	3,103 (6.5)	3,350 (7.0)	2,004 (6.7)	951 (6.3)	735 (5.4)	
BMI, kg/m^2^	16.7 ± 1.5	22.2 ± 1.8	27.3 ± 1.4	32.2 ± 1.4	37.1 ± 1.4	45.9 ± 5.0	<0.001
Heart rate, bpm	106 ± 32	101 ± 33	98 ± 33	98 ± 32	99 ± 32	100 ± 32	<0.001
Mean blood pressure, mmHg	81 ± 41	83 ± 41	86 ± 42	88 ± 43	89 ± 44	89 ± 45	<0.001
Severity score							
APACHE score	52 (34–71)	49 (31–67)	47(30–64)	45(30–64)	45(29–64)	46(29–64)	<0.001
GCS	14 (11–15)	15 (12–15)	15 (12–15)	15 (12–15)	15 (12–15)	15 (12–15)	<0.001
Primary reason of ICU admission
Cardiovascular disease, *n* (%)	1,744 (24.7)	15,694 (32.9)	19,504 (41.0)	12,877 (43.2)	6,173 (41.0)	4,850 (35.5)	<0.001
Respiratory disease, *n* (%)	2,053 (29.1)	8,772 (18.4)	7,125 (15.0)	4,524 (15.2)	2,581 (17.2)	2,942 (21.5)	<0.001
Digestive disease, *n* (%)	834 (11.8)	5,553 (11.6)	5,096 (10.7)	2,841 (9.5)	1,435 (9.5)	1,157 (8.5)	<0.001
Genitourinary disease, *n* (%)	365 (5.2)	2,438 (5.1)	2,239 (4.7)	1,547 (5.2)	868 (5.8)	965 (7.1)	<0.001
Neurological disease, *n* (%)	474 (6.7)	3,181 (6.7)	2,964 (6.2)	1,784 (6.0)	878 (5.8)	764 (5.6)	<0.001
Endocrine disease, *n* (%)	363 (5.1)	2,266 (4.7)	1,519 (3.2)	768 (2.6)	365 (2.4)	341 (2.5)	<0.001
Trauma, *n* (%)	221 (3.1)	2,323 (4.9)	1,984 (4.2)	1,085 (3.6)	379 (2.5)	277 (2.0)	<0.001
Other infectious disease, *n* (%)	296 (4.2)	1,793 (3.8)	1,667 (3.5)	1,155 (3.9)	662 (4.4)	778 (5.7)	<0.001
Other disease, *n* (%)	717 (11.1)	5,748 (12.0)	5,489 (11.5)	3,237 (10.8)	1,701 (11.3)	1,584 (11.6)	<0.001
Pre-admission comorbidities							
Coronary artery disease, *n* (%)	966 (13.7)	8,272 (17.3)	9,524 (20.0)	6,078 (20.4)	3,009 (20.0)	2,316 (17.0)	<0.001
Stroke/TIA, *n* (%)	687 (9.7)	4,709 (9.9)	4,710 (9.9)	2,704 (9.1)	1,235 (8.2)	1,031 (7.5)	<0.001
Diabetes mellitus, *n* (%)	572 (8.1)	4,674 (9.8)	5,291 (11.1)	4,221 (14.2)	2,711 (18.0)	3,007 (22.0)	<0.001
Hypertension, *n* (%)	2,522 (35.7)	19,372 (40.6)	21,825 (45.9)	14,641 (49.1)	7,685 (51.1)	7,117 (52.1)	<0.001
Congestive heart failure, *n* (%)	724 (10.2)	5,769 (12.1)	6,058 (12.7)	4,267 (14.3)	2,468 (16.4)	2,806 (20.5)	<0.001
Peripheral arterial disease, *n* (%)	306 (4.3)	2087 (4.4)	2,150 (4.5)	1,266 (4.2)	632 (4.2)	526 (3.9)	0.024
Chronic obstructive pulmonary disease, *n* (%)	1,527 (21.6)	6,474 (13.6)	5,533 (11.6)	3,646 (12.2)	2,132 (14.2)	2,399 (17.6)	<0.001
Renal dysfunction, *n* (%)	662 (9.4)	5,177 (10.8)	5,308 (11.2)	3,467 (11.6)	1866 (12.4)	1860 (13.6)	<0.001
Therapeutics							
Mechanical ventilation, *n* (%)	1,800 (25.5)	10,748 (22.5)	10,844 (22.8)	7,301 (24.5)	4,050 (26.9)	4,395 (32.2)	<0.001
Dialysis, *n* (%)	280 (4.0)	1927 (4.0)	1715 (3.6)	1,056 (3.5)	541 (3.6)	467 (3.4)	<0.001
Vasoactive drugs, *n* (%)	259 (3.7)	1,686 (3.5)	1760 (3.7)	1,042 (3.5)	526 (3.5)	481 (3.5)	0.624
Admission source							<0.001
Emergency department, *n* (%)	3,926 (55.6)	25,341 (53.1)	23,582 (49.6)	13,979 (46.9)	7,028 (46.7)	6,592 (48.3)	
Acute care/floor, *n* (%)	1,381 (19.5)	8,013 (16.8)	7,598 (16.0)	4,838 (16.2)	2,609 (17.3)	2,584 (18.9)	
Other, *n* (%)	1760 (24.9)	14,414 (30.2)	16,407 (34.5)	11,001 (36.9)	5,405 (35.9)	4,482 (32.8)	
Geographic location							<0.001
Midwest, *n* (%)	2,110 (29.9)	15,092 (31.6)	15,825 (33.3)	10,475 (35.1)	5,551 (36.9)	5,237 (38.3)	
South, *n* (%)	2,280 (32.3)	14,262 (29.9)	14,127 (29.7)	8,822 (29.6)	4,345 (28.9)	4,076 (29.8)	
West, *n* (%)	1,400 (19.8)	9,802 (20.5)	9,694 (20.4)	5,700 (19.1)	2,706 (18.0)	2,250 (16.5)	
Northeast, *n* (%)	451 (6.4)	3,122 (6.5)	3,156 (6.6)	2,130 (7.1)	1,132 (7.5)	1,025 (7.5)	
Other, *n* (%)	826 (11.7)	5,490 (11.5)	4,785 (10.1)	2,691 (9.0)	1,308 (8.7)	1,070 (7.8)	
Hospital discharge year							0.703
2014, *n* (%)	3,348 (47.4)	22,416 (46.9)	22,364 (47.0)	14,136 (47.4)	7,023 (46.7)	6,417 (47.0)	
2015, *n* (%)	3,719 (52.6)	25,352 (53.1)	25,223 (53.0)	15,682 (52.6)	8,019 (53.3)	7,241 (53.0)	
Length of stay, days	5.1 (2.8–8.9)	4.8 (2.5–8.3)	4.7 (2.5–8.1)	4.8 (2.6–8.2)	4.9 (2.6–8.7)	5.1 (2.8–9.2)	<0.001
All-cause death, *n* (%)	1,016 (14.4)	4,830 (10.1)	4,007 (8.4)	2,329 (7.8)	1,248 (8.3)	1,138 (8.3)	<0.001
Cardiovascular death, *n* (%)	289 (4.1)	1,641 (3.4)	1,654 (3.5)	1,008 (3.4)	523 (3.5)	450 (3.3)	0.040
Infectious-cause death, n (%)	385 (5.4)	1,560 (3.3)	1,112 (2.3)	627 (2.1)	311 (2.1)	313 (2.3)	<0.001
Other-cause death, *n* (%)	342 (4.8)	1,629 (3.4)	1,241 (2.6)	694 (2.3)	414 (2.8)	375 (2.7)	<0.001

Values are mean (standard deviation), median (inter-quartile range) or number (percentage). APACHE, acute physiology, age and chronic health evaluation. GCS, glasgow coma score.

### Obesity and all-cause mortality

**Figure 2:**
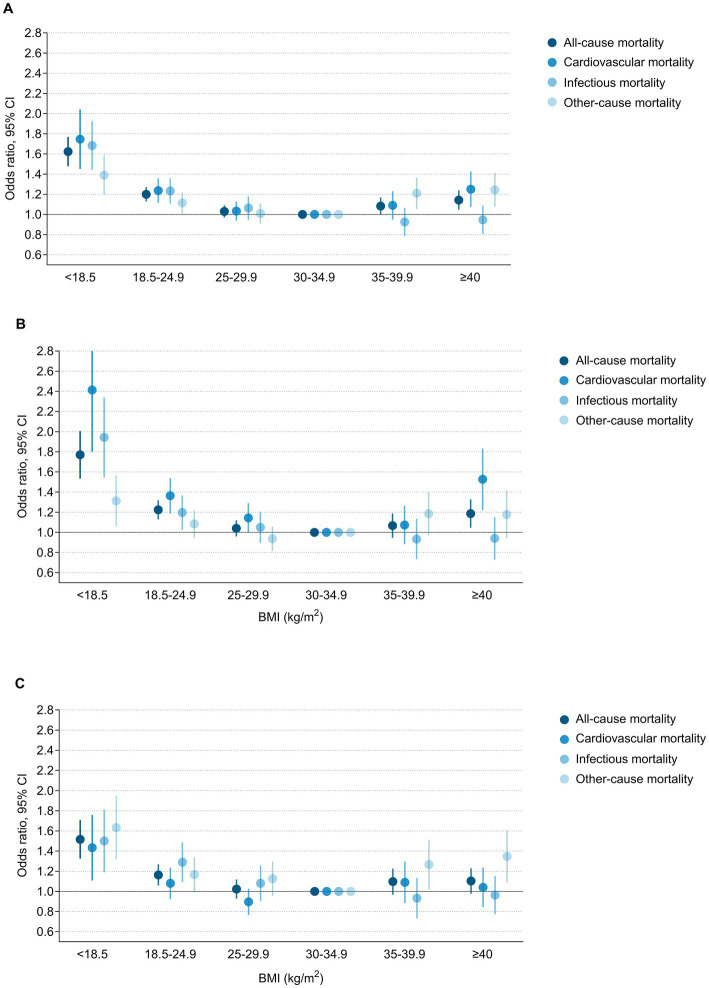
Multivariable adjusted odds ratios for all-cause and cause-specific mortality according to BMI on a categorical scale among **(A)** Overall population, **(B)** Men and **(C)** Women. Odds ratios and 95% confidence intervals were from multivariable adjusted logistic regression model.

With a multivariable logistic regression model, the association between BMI on a categorical scale and all-cause mortality was U-shaped in the general population and in men, with both a low and high BMI correlated with a greater risk of mortality. However, a reverse J-shaped association was noted in women, with only a low BMI increasing the risk of mortality. Underweight individuals had the highest mortality in the overall population (OR 1.62; 95% CI: 1.48–1.77), followed by those with normal weight (OR 1.20; 1.13–1.27), and the lowest mortality was observed among class I obese individuals. Underweight and normal weight men had corresponding odds ratios of 1.76 (95% CI: 1.54–2.01) and 1.22 (95% CI: 1.13–1.32) compared with class I obese men, respectively Similarly, underweight and normal weight among women were also independently associated with all-cause mortality, with odds ratios of 1.51 (95% CI: 1.33–1.71) and 1.16 (95% CI: 1.06–1.27), respectively, after adjustment for potential confounders. In the class III obese category, the multivariable adjusted odds ratios for all-cause mortality were 1.14 (95% CI: 1.05–1.24) for the overall population, 1.18 (95% CI: 1.05–1.33) for men and 1.10 (95% CI: 0.98–1.23) for women (Figure 2; Supplementary Table 1).

**Figure 3:**
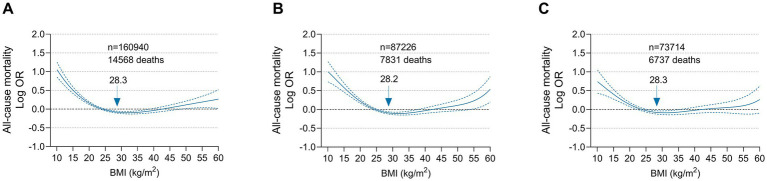
Multivariable adjusted odds ratios for all-cause mortality according to BMI on a continuous scale among **(A)** Overall population, **(B)** Men and **(C)** Women. Odds ratios (solid line) and 95% confidence intervals (dashed lines) were from cubic spline curves based on the generalized additive model. Arrows indicate BMI associated with the lowest mortality.

Based on the cubic spline curve, the association between BMI on a continuous scale and all-cause mortality was also U-shaped in the general population and in men, whereas it was reverse J-shaped in women. The risk inflection point correlated with the lowest all-cause mortality was 28.3 kg/m^2^ in the general population, 28.2 kg/m^2^ in men and 28.3 kg/m^2^ in women (Figure 3). Among the overall population, men and women with a BMI below the risk inflection point accounted for 54.6% (87906), 54.9% (47922) and 53.5% (39401), respectively. Before the corresponding risk inflection points, for every 5 kg/m^2^ decrease in BMI, the risk of all-cause mortality increased by 18% in the whole population, 21% in men and 15% in women. After the inflection points, the risk of death plateaued. For every 5 kg/m^2^ increase in BMI, the risk of all-cause mortality in the whole population, men and women increased by only 1% (Supplementary Table 2).

In stratified analysis, no clear evidence of a statistical interaction between BMI category and stratified variables on all-cause mortality was found. Compared with the class I obesity category, both the underweight category and normal weight category were independently associated with higher all-cause mortality, although the CI risk estimates were slightly wider in certain groups due to the relatively small number of individuals and events (Supplementary Table 3).

### Obesity and cause-specific mortality

**Figure 4:**
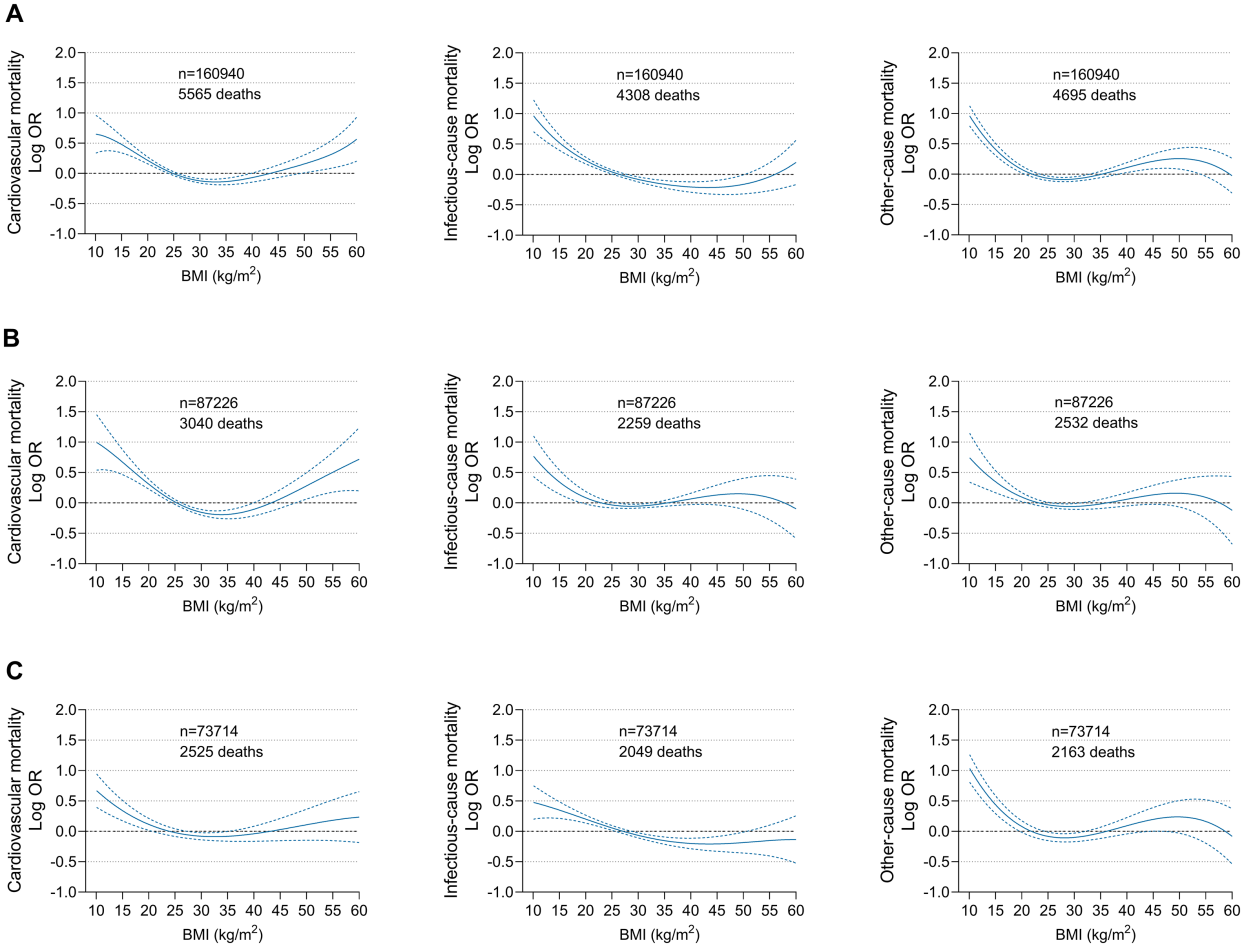
Multivariable adjusted odds ratios for cause-specific mortality according to BMI on a continuous scale among **(A)** Overall population, **(B)** Men and **(C)** Women. Odds ratios (solid line) and 95% confidence intervals (dashed lines) were from cubic spline curves based on the generalized additive model.

A U-shaped association between BMI and cardiovascular mortality was observed in the general population and in men, while a reverse J-shaped association was noted in women. Low BMI was consistently associated with increased cardiovascular mortality in the overall population and both sexes. However, the association between class III obesity and cardiovascular mortality was more pronounced among men (OR 1.51; 95% CI: 1.23–1.84) than among women (OR 1.03; 95% CI: 0.85–1.24) (P for interaction 0.0046) (Supplementary Figure 1). Regarding infectious disease mortality, there was a consistent monotonic decreased risk with increasing BMI in the general population and both sexes. Low BMI was strongly associated with an increased risk of infectious disease mortality, while high BMI was not related to it. The association between BMI and other-cause mortality exhibited U-shaped in the general population and in women but reverse J-shaped in men. Contrary to cardiovascular death, the relationship between class III obesity and other-cause mortality was significant among women (OR 1.33; 95% CI: 1.10–1.61) but not significant among men (OR 1.16; 95% CI: 0.95–1.42). Moreover, class II obese women also had an increased risk of other-cause mortality (OR 1.25; 95% CI: 1.03–1.52) (Figure 2; Supplementary Table 4). These findings were reconfirmed by a cubic spline model with BMI as a continuous variable (Figure 4).

### Obesity and specific disease-related mortality

**Figure 5:**
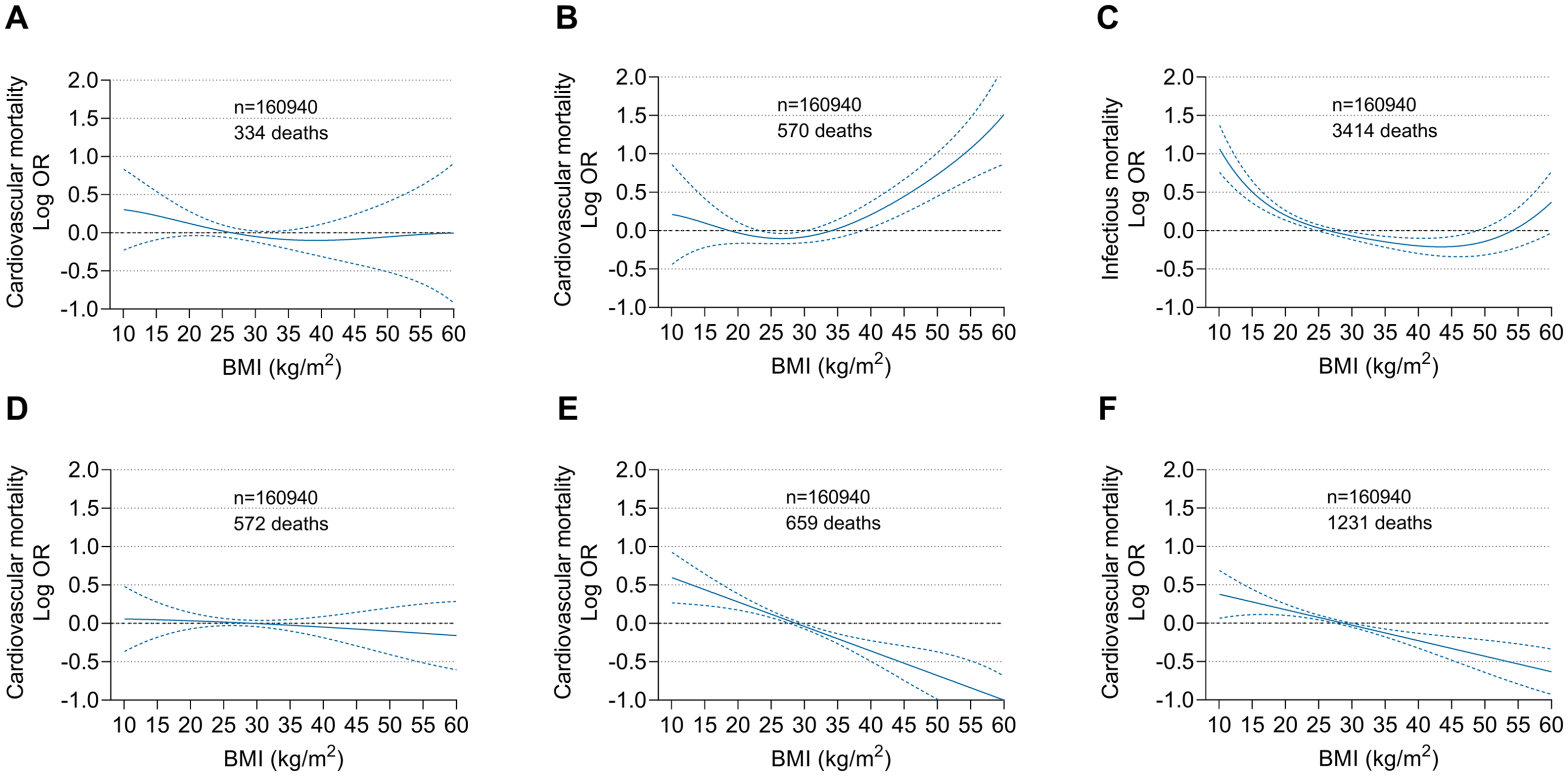
Multivariable adjusted odds ratios for specific disease related mortality according to BMI on a continuous scale. **(A)** Myocardial infarction, **(B)** Heart failure, **(C)** Sepsis, **(D)** Ischaemic stroke, **(E)** Intracranial hemorrhage and **(F)** Ischaemic and hemorrhagic stroke related mortality among the overall population. Odds ratios (solid line) and 95% confidence intervals (dashed lines) were from cubic spline curves based on the generalized additive model.

Regarding fatal myocardial infarction and fatal ischaemic stroke, no significant association was found between BMI and disease-related death. A J-shaped association between BMI and fatal heart failure-related death was observed, with a plateau at approximately a BMI of 25 to 30 kg/m^2^. When BMI exceeded this plateau, the risk of heart failure-related death increased significantly. There was a reverse J-shaped relationship for sepsis-related death, with a low BMI associated with high mortality, whereas a high BMI was not. A strongly monotonic decreased risk for intracranial haemorrhage-related death with increasing BMI was detected, and this significant negative correlation dominated the association between BMI and all stroke deaths, including ischaemic and haemorrhagic stroke (Figure 5).

### Sensitivity analysis

To evaluate the possible impact of reverse causality from severe illness, we examined the association between BMI and risk of mortality by excluding deaths that occurred within the first 48 h of ICU entry. The overall odds ratio was similar, only slightly attenuated (Supplementary Figure 2; Supplementary Table 5). The results from complete case analyses that included only individuals with complete data on all covariates were consistent with those of the main analysis, and the findings were greatly similar for men and women separately (Supplementary Figure 3; Supplementary Table 6). Finally, the result from Kaplan–Meier survival analysis considering mortality as a time-to-event variable with length of ICU stay as the timescale was also consistent with that of the primary analysis (Supplementary Figure 4).

## Discussion

In a large multicentre ICU cohort, we found a striking U-shaped or reverse J-shaped association between BMI and all-cause and cause-specific mortality among critically ill men and women, independent of obesity-related comorbidities and other potential confounding factors. Both underweight and normal weight individuals had a greater risk of death than their obese counterparts. These findings suggest that obesity exerts a protective effect on all-cause and cause-specific mortality among men and women, consistent with the obesity paradox. However, this protective effect appears not to extend to individuals with severe obesity (class III obesity). The relationship between severe obesity and cardiovascular mortality diverged between men and women. The current results confirm that the obesity paradox remains apparent among critically ill patients, but it is not applicable to severely obese patients. There is a sex difference in the impact of severe obesity on cause-specific mortality. These findings provide more information for predicting disease prognosis and improving the quality of ICU management.

In the past 30 years, the prevalence of obesity and the burdens of obesity-related diseases have gradually increased globally. It is predicted that as the prevalence of obesity in the general population increases, the incidence of obesity among critically ill patients will also increase. A meta-analysis reported that approximately one-third of ICU patients were obese, and nearly 7% were morbidly obese (20). The high incidence of obesity in this study was consistent with previous results, with 36.3% of obese patients and 8.5% of severely obese patients. The association pattern of obesity and adverse outcomes has been investigated in some relatively small studies in the critical care field, with the obesity paradox existing in short-term and long-term all-cause mortality (23, 24). Akinnusi et al. reported a U-shaped correlation between BMI and mortality, with worse survival among underweight (BMI <18.5 kg/m^2^) and morbidly obese (>40 kg/m^2^) patients (20). Oliveros et al. found a lower mortality among obese patients (BMI 30.0–39.9 kg/m^2^) but not among morbidly obese patients (BMI > 40 kg/m^2^) when using normal weight patients as a reference (25). In alignment with previous studies, this analysis showed that obese individuals had a better 30-day survival rate, although they had a higher incidence of clinical comorbidities, including hypertension, diabetes mellitus, heart failure and renal insufficiency, and the differences in these comorbidity patterns may be the major confounding factors affecting the clinical prognosis. Underweight individuals had an approximately 1.7-fold increased risk, and normal weight individuals had a 1.2-fold increased risk of all-cause mortality compared to their class I obese counterparts, which was noted among the overall population, among men and women. Among class III obese individuals, increased all-cause mortality was observed among men but not among women, resulting in a U-shaped association among the overall population and among men and a reverse J-shaped association among women. These findings support the existence of the obesity paradox, but the survival benefit does not extend to class III obese individuals, especially men. Furthermore, extremely close BMI inflection points for all-cause mortality were generated by the cubic spline curves, with 28.3 kg/m^2^ for the whole population, 28.2 kg/m^2^ for males and 28.3 kg/m^2^ for females. There are several potential explanations for the obesity paradox. First, adipocytes positively regulate worsening inflammatory processes by secreting immunomodulatory substances such as leptin and interleukin-10, thereby improving survival during severe illness (26). Second, high cholesterol and lipoprotein levels in obese individuals may provide the precursors for adrenal steroid hormone synthesis to combat lethal stress (27). Third, adipose tissue also affords important nutritional reserves for critically ill patients with highly catabolic status and negative energy balance (28). Fourth, underweight individuals are usually more vulnerable and have less positive responses to supportive therapy (29). Finally, disparities in medical care may also lead to survival differences. Due to subconsciously entrenched concerns about obesity, obese patients often receive earlier and more aggressive management and are assigned closer monitoring, higher care standards and a lower threshold for transfer to the ICU. Indeed, this analysis showed that obese patients had higher rates of mechanical ventilation usage, partly reflecting more aggressive interventions.

The association pattern between BMI and cardiovascular death was largely consistent with that of all-cause death, supporting the obesity paradox. Notably, an obviously increased cardiovascular mortality was found among class III obese men but not women. Sex hormones may play an important role in determining fat mass and distribution. Oestrogen increases fat deposition, while testosterone inhibits fat deposition, so men tend to have less fat mass than women (30). In addition, because oestrogen blocks the androgen effect by downregulating the androgen receptor, women tend to accumulate more subcutaneous fat but less visceral fat than men (31). Visceral fat appears to be the major pathogenic fat depot associated with cardiovascular and metabolic alterations. Its proinflammatory, prothrombotic and low-fibrinogen milieus have a negative impact on cardiovascular protection and metabolic regulation, while subcutaneous fat acts more as a metabolic reserve, helping other tissues defend against lipotoxicity (32). These mechanisms could partly explain our findings that extremely obese men still face an increased risk of cardiovascular death, while women may be exempt due to the heterogeneity in adipose distribution. A recent study of a large cohort of women with coronary artery disease treated with drug-eluting stents also showed that the adjusted risk estimates for cardiovascular mortality among severely obese women were not statistically significant (33), which was in line with our findings. In addition, a cardiovascular magnetic resonance study explained this issue from an imaging perspective, that is, there was a sex-specific difference in left ventricular remodelling among obese subjects (34). Men predominantly exhibited concentric hypertrophy, while women presented a combination of eccentric and concentric hypertrophy. Concentric hypertrophy is proven to be more closely associated with cardiovascular mortality than eccentric hypertrophy.

The obesity paradox among patients with pneumonia and sepsis has been observed, despite evidence supporting that obesity impairs the immune response and increases susceptibility to infection (35). In this study, there was a consistent reverse J-shaped association between BMI and infectious disease mortality across the whole population, men and women. Only underweight and normal weight individuals had an increased risk, and when BMI exceeded 25 kg/m^2^, the risk of infectious disease death no longer increased but tended to decrease. The potential link between obesity and lower infectious disease mortality may be related to adipocytes positively regulating worsening inflammatory processes, high lipid levels neutralizing circulating endotoxin, and adipocytes providing adrenal steroid synthesis precursors and energy storage (26–28).

Other-cause deaths in this study included trauma, cancer, and uncommon disease-related deaths. Due to the relatively small number of events, separate analysis of a single disease could not be performed. Obesity had a protective effect on risk-adjusted mortality among individuals who died of noncardiovascular and noninfectious causes. However, this protective effect did not extend to severely obese individuals. Severely obese women remained at significantly increased risk of death compared with their mildly obese counterparts.

Previous studies have shown that obesity has a contradictory protective effect on heart failure (14, 15). However, it has also been suggested that the obesity paradox disappears after adjusting for B-type natriuretic peptide levels (36). We found that obesity was positively correlated with 30-day mortality among patients with acute heart failure. One possible explanation is that high BMI in the acute phase may be due to fluid retention rather than fat accumulation, affecting short-term prognosis, while cardiac cachexia and tissue hypoperfusion may contribute to worse long-term prognosis. These findings indicate that obesity may have different impacts on short-term and long-term prognoses among patients with heart failure. A prior heart failure study also showed that high BMI had a protective effect on 1-year mortality but not on 30-day mortality (37). The monotonous negative correlation between BMI and intracranial haemorrhage-related death was in line with expectations. A prospective study among 1.3 million British women revealed a robust relationship between low BMI and haemorrhagic stroke-related death (38). The trend in sepsis-related death was consistent with that of infectious disease death, with sepsis-related deaths accounting for 79.3% (3,414 of 4,308) of infectious disease deaths.

The unequal presentation of the obesity paradox between sexes has been reported. Studies among patients with heart failure and cardiogenic shock showed that the obesity paradox occurred only among men and not among women (14, 39). Clark et al. found that both women and men with systolic heart failure were affected by the obesity paradox (15). Our study showed that the obesity paradox apparently existed among both men and women, which was identified by BMI as a categorical variable and a continuous variable. However, the impact of the obesity paradox did not extend to severely obese individuals, and there was a sex difference between extremely high BMI and cause-specific mortality. Severe obesity increased cardiovascular deaths among men and increased other-cause deaths among women, leading to increased cardiovascular deaths and other-cause deaths among severely obese individuals in the overall population. According to these findings, we have several considerations. First, BMI may not be a perfect anthropometric indicator for characterizing obesity due to its inherent limitations in assessing body composition and fat distribution. Obese individuals may have increased lean mass or more favourable subcutaneous fat distribution than visceral fat distribution, and these clinical phenotypes may confuse the findings of the obesity paradox. However, there is no corresponding suspicion when BMI is considered a risk predictor for pathophysiological disorders. Therefore, the defects of evaluation indicators cannot completely deny the obesity paradox. The obesity paradox may indicate a lack of comprehension of the complex pathophysiological link between obesity and clinical outcomes, requiring further study. Second, reports on the obesity paradox have brought a confusing message to clinicians and policy-makers, leading to misguided healthy lifestyle management. However, given that obesity is a significant contributor to various pathophysiological dysfunctions and causes a substantial multimorbidity burden, the debate of the obesity paradox should not reduce efforts to control obesity while awaiting further evidence. Moreover, our study also showed that severe obesity led to worse survival. Third, the current results are generally consistent with and further extend previous reports on the obesity paradox in various clinical milieus. Although this paradox exists among both men and women, it cannot be extended to severely obese individuals. The increased cardiovascular death among severely obese men drove the increased all-cause mortality risk, while the increased other-cause death among severely obese women led to an upward trend of all-cause mortality risk. However, infectious deaths did not appear to be involved. Therefore, in addition to focusing on the greater risk among underweight and normal weight patients, clinicians should pay special attention to the risk of cardiovascular death among severely obese men and other-cause death among severely obese women and manage potential complications and risk factors that may compromise survival. Finally, the association between BMI and disease-specific mortality underscores that the impact of obesity on mortality may be subdivided and cannot be simply summarized in terms of the obesity paradox. Developing more accurate and targeted predictors to provide precise and personalized assessments is needed for future research.

### Strengths and limitations

This study included more than 160,000 ICU patients from a contemporary multicentre database. It was heterogeneous in terms of disease composition, ICU type and admission source, yielding a certain extrapolation validity for the study results. The model was extensively adjusted for confounding factors and had significant statistical power. Moreover, we extended the existing view of the obesity paradox and posited that it cannot be extended to severely obese individuals and that there was a sex difference in the impact of obesity on cause-specific mortality. Several limitations need to be considered. First, given that the retrospective design is inherently limited, we could not prove a causal relationship between obesity and mortality. Second, we broadly adjusted for confounding factors in multivariate analysis, including not only disease type and clinical comorbidities but also mechanical ventilation, dialysis, and vasoactive drug usage. Obesity leads to increased use and duration of mechanical ventilation, requires more frequent dialysis to achieve sufficient clearance, and affects the titration of vasoactive drugs, which may have an impact on mortality. However, obesity may be a net result of complex interactions between genetic, behavioural and environmental factors. Residual confounding factors, including dietary habits, smoking history, alcohol consumption, physical activity, income and socioeconomic status, may be involved, which were not extracted from the database. Third, we did not have any information about abdominal obesity or adipose distribution, such as waist circumference and waist-hip ratio, which may have an additional impact on the outcomes. Fourth, the subset of severely obese individuals was relatively small in number, which may have limited the statistical power of this group. Fifth, this study has a large heterogeneity in ethnic composition, and the majority of the cohort is Caucasians, accounting for 77% of the total population, which limits the extrapolation of the current results to other ethnic populations. Finally, given the regional differences in the definition of obesity based on BMI, a large proportion of individuals from the United States and European countries may restrict the extrapolation of these findings.

## Conclusion

With the rapid development of the global economy and the general improvement of living standards, obesity is likely to become an increasingly prominent concern in the ICU. Our study provides new evidence on the obesity paradox, which is a well-known phenomenon in a variety of disease entities and is still evident among critically ill patients. Although the protective effect of obesity on all-cause and cause-specific mortality is largely consistent among men and women, this effect cannot be extended to severely obese individuals. Special attention needs to be paid to cardiovascular death risk among severely obese men and other-cause death risk among severely obese women.

## Data availability statement

Publicly available datasets were analyzed in this study. This data can be found at: https://www.physionet.org/content/eicu-crd-demo/2.0.1/.

## Ethics statement

The studies involving human participants were reviewed and approved by the institutional review board of the Massachusetts Institute of Technology. The ethics committee waived the requirement of written informed consent for participation.

## Author contributions

SL has fully obtained all the data and takes responsibility for the integrity of the data and the accuracy of the analysis. SL and HL contributed to the concept and study design. SL and WZ contributed to the acquisition, statistical analysis, and interpretation of the data. SL and ZF contributed to the drafting of the manuscript. All authors contributed to the article and approved the submitted version.

## Conflict of interest

The authors declare that the research was conducted in the absence of any commercial or financial relationships that could be construed as a potential conflict of interest.

## Publisher’s note

All claims expressed in this article are solely those of the authors and do not necessarily represent those of their affiliated organizations, or those of the publisher, the editors and the reviewers. Any product that may be evaluated in this article, or claim that may be made by its manufacturer, is not guaranteed or endorsed by the publisher.

## Footnotes

^1^ http://www.r-project.org

^2^ http://www.empowerstats.com
