# Supplementary material for: Impact of obesity on all-cause and cause-specific mortality among critically ill men and women: a cohort study on the eICU database
Source: Front Nutr. 2023 Apr 21;10:1143404. doi: 10.3389/fnut.2023.1143404 (PMC10160369; doi:10.3389/fnut.2023.1143404)
Supplement: Supplementary file 1 [file Data_Sheet_1.PDF]

Supplementary Material

Impact of obesity on all-cause and cause-specific mortality among critically ill men and women: A cohort study on the eICU database

Shan Li[*], **Wei Zhang**[†], **Hongbin Liu**

* **Correspondence**: Shan Li. lishan301301@163.com

[†] **First authorship**: Wei Zhang

1 Supplementary Data

Supplementary Material should be uploaded separately on submission. Please include any supplementary data, figures and/or tables.

Supplementary material is not typeset so please ensure that all information is clearly presented, the appropriate caption is included in the file and not in the manuscript, and that the style conforms to the rest of the article.

2 Supplementary Figures and Tables

For more information on Supplementary Material and for details on the different file types accepted, please see here.

2.1 Supplementary Figures

Supplementary Figure 1. Multivariable adjusted odds ratios for all-cause and cause-specific mortality for underweight and class III obesity categories among men and women. (A) Underweight (<18.5 kg/m^2) vs. class I obesity (30.0-34.9 kg/m^2) and (B) Class III obesity (≥40 kg/m^2) vs. class I obesity (30.0-34.9 kg/m2).

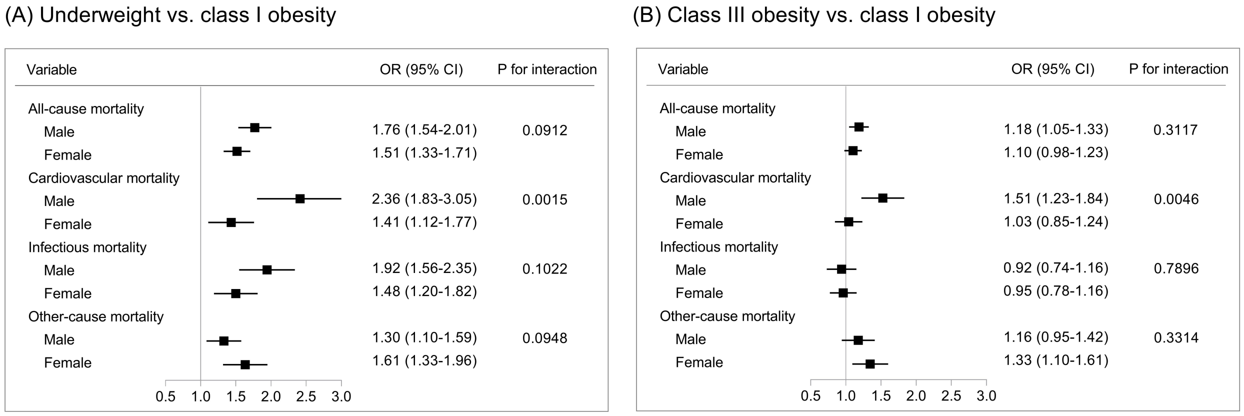

Supplementary Figure 2. Multivariable adjusted odds ratios for all-cause and cause-specific mortality according to BMI on a continuous scale among (A) Overall population, (B) Men and (C) Women after exclusion of deaths within 48 hours of admission

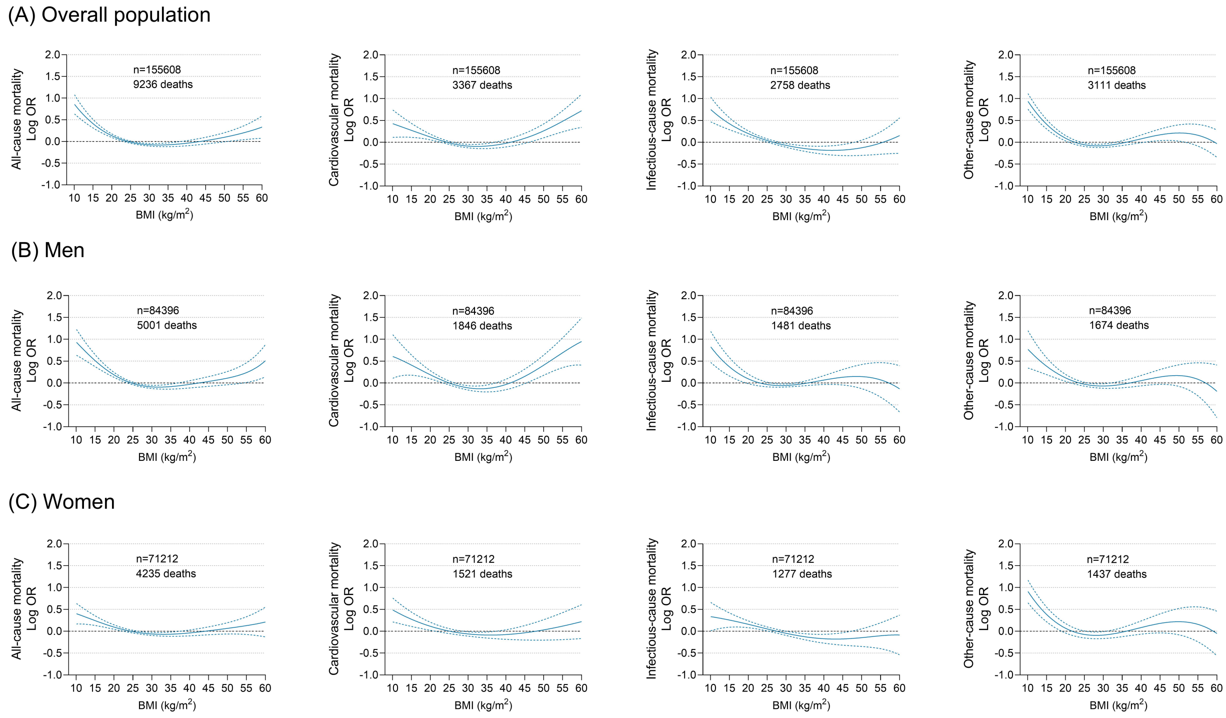

Supplementary Figure 3. Multivariable adjusted odds ratios for all-cause and cause-specific mortality according to BMI on a continuous scale among (A) Overall population, (B) Men and (C) Women with complete-case analysis

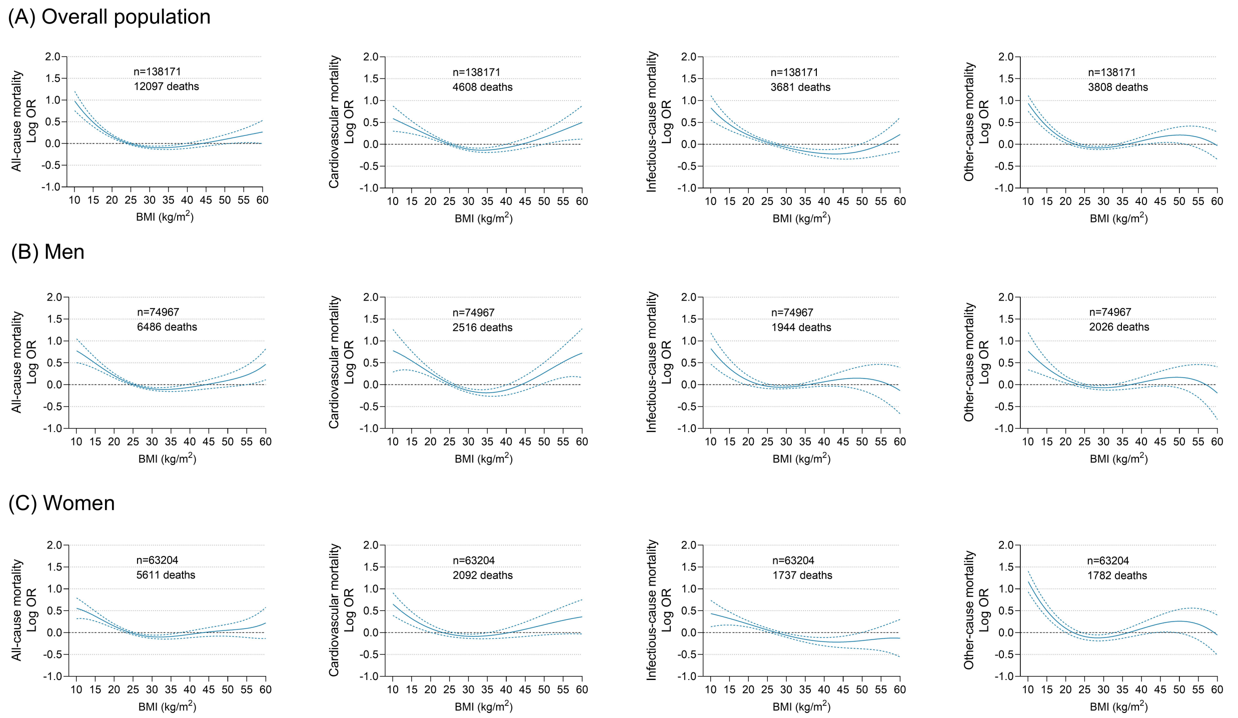

Supplementary Figure 4. Kaplan-Meier curve for cumulative incidence of all-cause and cause-specific mortality by BMI categories among (A) Overall population, (B) Men and (C) Women. Analyses were adjusted for age, ethnicity, heart rate, mean blood pressure, APACHE score, GCS, primary reason for admission (cardiovascular disease, respiratory disease, digestive disease, genitourinary disease, neurological disease, endocrine disease, trauma, other infectious disease), preadmission comorbidities (coronary artery disease, stroke/transient ischemic attacks, diabetes mellitus, hypertension, congestive heart failure, peripheral artery disease, chronic obstructive pulmonary disease, renal dysfunction), invasive mechanical ventilation, dialysis, vasoactive drugs, admission source, geographic location and discharge year.

(A) Overall population

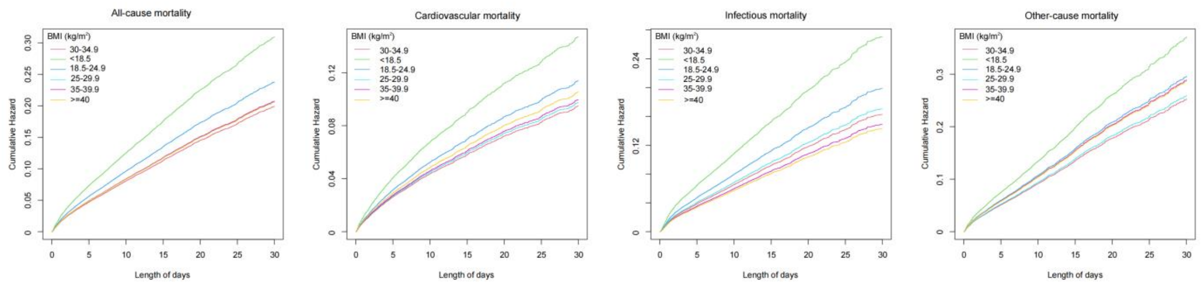

(B) Men

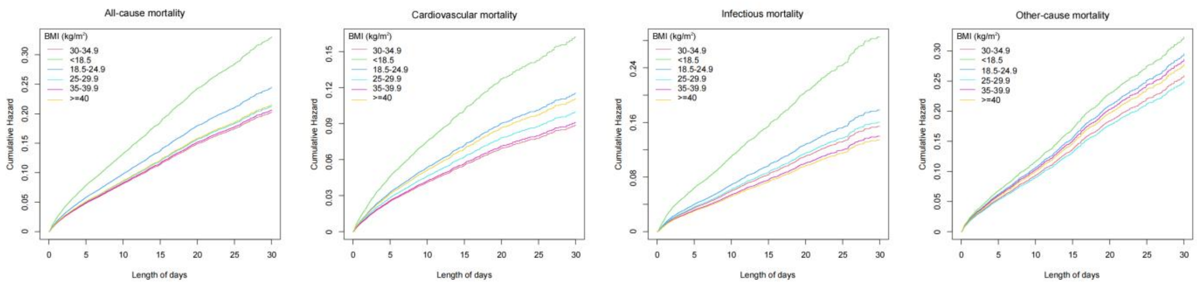

(C) Women

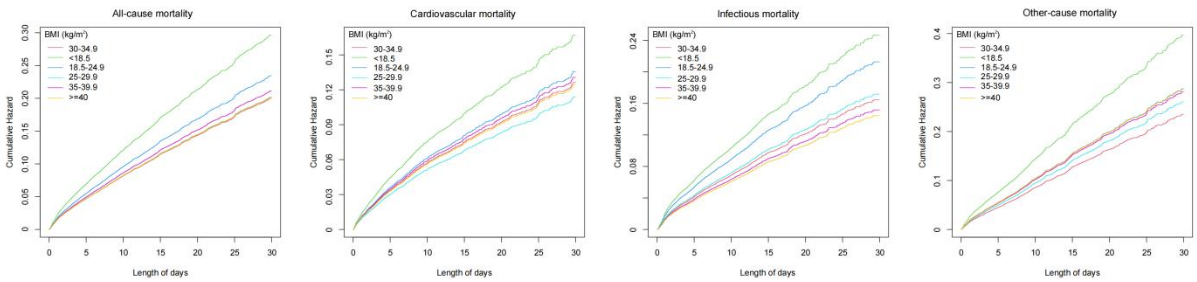

Supplementary Table 1. Univariable and multivariable adjusted odds ratios for all-cause mortality according to BMI on a categorical scale. Model I unadjusted, model II adjusted for age, sex and ethnicity, model III adjusted for age, sex, ethnicity, heart rate, mean blood pressure, APACHE score, GCS, primary reason for admission (cardiovascular disease, respiratory disease, digestive disease, genitourinary disease, neurological disease, endocrine disease, trauma, other infectious disease), preadmission comorbidities (coronary artery disease, stroke/transient ischemic attacks, diabetes mellitus, hypertension, congestive heart failure, peripheral artery disease, chronic obstructive pulmonary disease, renal dysfunction), mechanical ventilation, dialysis, vasoactive drugs, admission source, geographic location and discharge year.

BMI category	Underweight <18.5 kg/m²	normal weight 18.5-24.9 kg/m²	Overweight 25-29.9 kg/m²	class I obesity 30-34.9 kg/m²	class II obesity 35-39.9 kg/m²	class III obesity ≥40 kg/m²
	OR (95% CI)	OR (95% CI)	OR (95% CI)	Reference	OR (95% CI)	OR (95% CI)
**Overall population**						
Model I	1.98 (1.83, 2.14)	1.33 (1.26, 1.40)	1.09 (1.03, 1.14)	1	1.07 (0.99, 1.15)	1.07 (1.00, 1.16)
Model II	1.88 (1.74, 2.04)	1.24 (1.18, 1.31)	1.03 (0.97, 1.08)	1	1.13 (1.05, 1.22)	1.24 (1.15, 1.33)
Model III	1.62 (1.48, 1.77)	1.20 (1.13, 1.27)	1.03 (0.97, 1.09)	1	1.08 (1.00, 1.17)	1.14 (1.05, 1.24)
**Men**						
Model I	2.13 (1.90, 2.38)	1.36 (1.27, 1.46)	1.08 (1.01, 1.16)	1	1.05 (0.95, 1.16)	1.13 (1.01, 1.26)
Model II	2.11 (1.88, 2.37)	1.28 (1.19, 1.37)	1.01 (0.94, 1.09)	1	1.11 (1.00, 1.22)	1.30 (1.17, 1.45)
Model III	1.76 (1.54, 2.01)	1.22 (1.13, 1.32)	1.04 (0.96, 1.12)	1	1.06 (0.95, 1.19)	1.18 (1.05, 1.33)
**Women**						
Model I	1.86 (1.67, 2.08)	1.28 (1.19, 1.39)	1.09 (1.01, 1.19)	1	1.08 (0.98, 1.20)	1.02 (0.92, 1.13)
Model II	1.71 (1.53, 1.91)	1.20 (1.11, 1.30)	1.05 (0.96, 1.13)	1	1.16 (1.04, 1.28)	1.18 (1.07, 1.31)
Model III	1.51 (1.33, 1.71)	1.16 (1.06, 1.27)	1.02 (0.93, 1.12)	1	1.09 (0.97, 1.23)	1.10 (0.98, 1.23)

Supplementary Table 2. Univariable and multivariable analyses for all-cause mortality with BMI below or above the risk inflection point. Model I unadjusted, model II adjusted for age, sex and ethnicity, model III adjusted for age, sex, ethnicity, heart rate, mean blood pressure, APACHE score, GCS, primary reason for admission (cardiovascular disease, respiratory disease, digestive disease, genitourinary disease, neurological disease, endocrine disease, trauma, other infectious disease), preadmission comorbidities (coronary artery disease, stroke/transient ischemic attacks, diabetes mellitus, hypertension, congestive heart failure, peripheral artery disease, chronic obstructive pulmonary disease, renal dysfunction), mechanical ventilation, dialysis, vasoactive drugs, admission source, geographic location and discharge year.

BMI (kg/m^2)	Individuals, n	Events (%)	Model I OR (95% CI)	Model II OR (95% CI)	Model III OR (95% CI)
**Overall population**					
Increase per kg/m^2 (<28.3)	87906	8706(9.9)	0.95 (0.94, 0.95)	0.94 (0.94, 0.95)	0.96 (0.95, 0.97)
Increase per 5 kg/m^2 (<28.3)	87906	8706(9.9)	0.76 (0.74, 0.79)	0.75 (0.73, 0.78)	**0.82 (0.79, 0.85)**
Increase per kg/m^2 (≥ 28.3)	73034	5862(8.0)	1.01 (1.00, 1.01)	1.02 (1.01, 1.02)	1.01 (1.01, 1.02)
Increase per 5 kg/m^2 (≥28.3)	73034	5862(8.0)	1.03 (1.01, 1.05)	1.08 (1.06, 1.10)	**1.05 (1.03, 1.08)**
**Men**					
Increase per kg/m^2 (<28.2)	47922	4734(9.9)	0.94 (0.93, 0.95)	0.93 (0.93, 0.94)	0.95 (0.94, 0.96)
Increase per 5 kg/m^2 (<28.2)	47922	4734(9.9)	0.74 (0.70, 0.77)	0.71 (0.68, 0.75)	**0.79 (0.75, 0.83)**
Increase per kg/m^2 (≥ 28.2)	39304	3097(7.9)	1.01 (1.00, 1.02)	1.02 (1.01, 1.03)	1.01 (1.01, 1.02)
Increase per 5 kg/m^2 (≥28.2)	39304	3097(7.9)	1.05 (1.02, 1.08)	1.11 (1.07, 1.14)	**1.06 (1.03, 1.10)**
**Women**					
Increase per kg/m^2 (<28.3)	39401	3935(10.0)	0.95 (0.94, 0.96)	0.96 (0.95, 0.97)	0.97 (0.96, 0.98)
Increase per 5 kg/m^2 (<28.3)	39401	3935(10.0)	0.79 (0.75, 0.83)	0.80 (0.76, 0.84)	**0.85 (0.80, 0.89)**
Increase per kg/m^2 (≥ 28.3)	34313	2802(8.2)	1.00 (1.00, 1.01)	1.01 (1.01, 1.02)	1.01 (1.00, 1.02)
Increase per 5 kg/m^2 (≥28.3)	34313	2802(8.2)	1.01 (0.98, 1.04)	1.06 (1.03, 1.09)	**1.05 (1.02, 1.09)**

Supplementary Table 3. Multivariable adjusted odds ratios for all-cause mortality according to BMI categories stratified by covariates. Model was adjusted for age, sex, ethnicity, heart rate, mean blood pressure, APACHE score, GCS, primary reason for admission (cardiovascular disease, respiratory disease, digestive disease, genitourinary disease, neurological disease, endocrine disease, trauma, other infectious disease), preadmission comorbidities (coronary artery disease, stroke/transient ischemic attacks, diabetes mellitus, hypertension, congestive heart failure, peripheral artery disease, chronic obstructive pulmonary disease, renal dysfunction), invasive mechanical ventilation, dialysis, vasoactive drugs, admission source, geographic location and discharge year.

	BMI, kg/m^2						P for interaction
Subgroup	<18.5	18.5-24.9	25.0-29.9	30.0-34.9	35.0-39.9	≥40	
	OR (95% CI)	OR (95% CI)	OR (95% CI)	reference	OR (95% CI)	OR (95% CI)	
**Age, years**							0.1679
<50	1.57 (1.18, 2.09)	0.92 (0.75, 1.11)	0.91 (0.75, 1.11)	1	1.12 (0.88, 1.43)	1.22 (0.96, 1.54)	
50-70	1.86 (1.59, 2.17)	1.21 (1.10, 1.34)	1.04 (0.94, 1.15)	1	1.05 (0.93, 1.19)	1.08 (0.96, 1.22)	
≥70	1.70 (1.51, 1.91)	1.36 (1.26, 1.47)	1.10 (1.01, 1.19)	1	1.05 (0.93, 1.18)	1.01 (0.88, 1.15)	
**Ethnicity**							0.2140

Caucasian	1.71 (1.55, 1.90)	1.27 (1.19, 1.35)	1.06 (0.99, 1.14)	1	1.06 (0.97, 1.16)	1.11 (1.01, 1.21)	
African American	1.75 (1.34, 2.28)	1.23 (1.02, 1.49)	1.08 (0.89, 1.31)	1	1.23 (0.97, 1.58)	1.19 (0.93, 1.51)	
Hispanic	1.67 (1.03, 2.73)	0.80 (0.59, 1.10)	0.80 (0.59, 1.09)	1	0.64 (0.40, 1.04)	0.79 (0.48, 1.29)	
Asian	1.05 (0.54, 2.05)	1.14 (0.67, 1.94)	0.83 (0.47, 1.47)	1	2.49 (1.07, 5.80)	1.36 (0.44, 4.23)	
Other/unknown	1.65 (1.11, 2.45)	1.09 (0.86, 1.38)	0.98 (0.77, 1.24)	1	1.09 (0.80, 1.50)	1.08 (0.76, 1.53)	
**Primary reason for ICU admission**							
**Cardiovascular disease**							0.0824
Yes	1.81 (1.53, 2.15)	1.28 (1.16, 1.41)	1.04 (0.95, 1.15)	1	1.08 (0.95, 1.23)	1.21 (1.05, 1.39)	
No	1.66 (1.49, 1.84)	1.20 (1.12, 1.29)	1.03 (0.96, 1.11)	1	1.08 (0.97, 1.20)	1.09 (1.00, 1.21)	
**Respiratory disease**							0.0590
Yes	1.74 (1.49, 2.03)	1.32 (1.18, 1.48)	1.08 (0.95, 1.22)	1	1.00 (0.85, 1.18)	1.06 (0.94, 1.20)	
No	1.58 (1.42, 1.77)	1.15 (1.08, 1.23)	1.02 (0.95, 1.09)	1	1.11 (1.01, 1.22)	1.20 (1.09, 1.33)	
**Digestive disease**							0.6755
Yes	1.77 (1.35, 2.31)	1.28 (1.07, 1.52)	1.03 (0.86, 1.24)	1	1.14 (0.89, 1.45)	1.21 (0.93, 1.58)	
No	1.68 (1.53, 1.85)	1.21 (1.14, 1.28)	1.03 (0.97, 1.10)	1	1.08 (0.99, 1.17)	1.12 (1.03, 1.23)	
**Neurological disease**							
Yes	1.45 (0.91, 2.31)	1.21 (0.88, 1.67)	1.14 (0.82, 1.58)	1	1.47 (0.96, 2.26)	1.54 (1.14, 2.68)	0.0977
No	1.72 (1.57, 1.88)	1.22 (1.15, 1.29)	1.03 (0.97, 1.10)	1	1.07 (0.99, 1.16)	1.11 (1.02, 1.21)	
**Genitourinary disease**							0.4147
Yes	1.24 (0.84, 1.83)	1.06 (0.83, 1.35)	0.86 (0.67, 1.12)	1	0.87 (0.61, 1.22)	1.10 (0.80, 1.52)	
No	1.71 (1.56, 1.88)	1.22 (1.15, 1.30)	1.04 (0.98, 1.11)	1	1.10 (1.01, 1.20)	1.14 (1.04, 1.24)	
**Trauma**							0.7895
Yes	2.06 (1.14, 3.71)	1.04 (0.73, 1.47)	0.95 (0.67, 1.35)	1	0.91 (0.52, 1.58)	1.20 (0.65, 2.20)	
No	1.68 (1.54, 1.84)	1.22 (1.15, 1.29)	1.03 (0.97, 1.10)	1	1.09 (1.00, 1.18)	1.13 (1.04, 1.23)	
**Preadmission comorbidities**							
**Coronary artery disease**							0.7642
Yes	1.65 (1.33, 2.05)	1.26 (1.11, 1.42)	0.99 (0.87, 1.12)	1	0.94 (0.78, 1.12)	1.20 (0.99, 1.45)	
No	1.72 (1.56, 1.90)	1.23 (1.16, 1.32)	1.06 (0.99, 1.14)	1	1.11 (1.02, 1.22)	1.09 (0.99, 1.20)	
**Diabetes mellitus**							0.2236
Yes	1.23 (0.85, 1.76)	1.08 (0.91, 1.28)	0.93 (0.79, 1.09)	1	0.95 (0.78, 1.15)	0.93 (0.77, 1.12)	
No	1.75 (1.59, 1.92)	1.26 (1.19, 1.34)	1.06 (1.00, 1.13)	1	1.10 (1.01, 1.20)	1.17 (1.06, 1.28)	
**Hypertension**							0.6779
Yes	1.75 (1.52, 2.01)	1.28 (1.18, 1.39)	1.06 (0.98, 1.16)	1	1.04 (0.93, 1.16)	1.09 (0.97, 1.23)	
No	1.66 (1.48, 1.87)	1.19 (1.10, 1.29)	1.02 (0.94, 1.11)	1	1.11 (0.99, 1.25)	1.14 (1.01, 1.28)	
**Ischemic stroke**							0.4517
Yes	1.86 (1.42, 2.43)	1.34 (1.12, 1.60)	1.01 (0.84, 1.22)	1	1.21 (0.94, 1.57)	1.04 (0.78, 1.39)	
No	1.69 (1.54, 1.86)	1.23 (1.16, 1.31)	1.05 (0.99, 1.12)	1	1.06 (0.97, 1.15)	1.11 (1.02, 1.22)	
**Chronic obstructive pulmonary disease**							0.1853
Yes	1.77 (1.46, 2.13)	1.18 (1.03, 1.37)	1.10 (0.95, 1.28)	1	0.96 (0.78, 1.16)	1.06 (0.87, 1.28)	
No	1.68 (1.52, 1.86)	1.25 (1.17, 1.33)	1.04 (0.97, 1.11)	1	1.10 (1.01, 1.20)	1.12 (1.02, 1.23)	
**Geographic region**							0.9571
Midwest	1.72 (1.46, 2.03)	1.27 (1.14, 1.41)	1.07 (0.96, 1.19)	1	1.07 (0.93, 1.23)	1.14 (0.99, 1.31)	
South	1.62 (1.38, 1.90)	1.20 (1.08, 1.34)	0.98 (0.88, 1.09)	1	1.09 (0.94, 1.25)	1.09 (0.93, 1.26)	

West	1.63 (1.33, 2.00)	1.19 (1.04, 1.36)	1.03 (0.90, 1.18)	1	1.08 (0.89, 1.30)	1.05 (0.86, 1.29)	
Northeast	1.72 (1.39, 2.12)	1.22 (1.06, 1.40)	1.14 (0.99, 1.31)	1	1.06 (0.87, 1.29)	1.16 (0.95, 1.43)	
**Treatment**							
**Vasoactive agent**							0.1566
Yes	1.70 (1.21, 2.38)	1.37 (1.12, 1.69)	1.02 (0.83, 1.26)	1	1.03 (0.78, 1.36)	1.46 (1.10, 1.93)	
No	1.68 (1.53, 1.84)	1.21 (1.14, 1.29)	1.04 (0.98, 1.11)	1	1.08 (0.99, 1.17)	1.08 (0.99, 1.18)	
**Mechanical ventilation**							0.6030
Yes	1.79 (1.56, 2.05)	1.32 (1.21, 1.44)	1.10 (1.01, 1.20)	1	1.09 (0.97, 1.22)	1.12 (1.00, 1.25)	
No	1.59 (1.42, 1.80)	1.17 (1.08, 1.27)	1.00 (0.92, 1.09)	1	1.08 (0.97, 1.21)	1.11 (0.98, 1.26)	
**Dialysis**							0.1072
Yes	1.45 (0.97, 2.17)	0.78 (0.61, 1.00)	0.79 (0.61, 1.02)	1	0.72 (0.51, 1.02)	0.90 (0.62, 1.29)	
No	1.71 (1.56, 1.87)	1.26 (1.19, 1.34)	1.06 (1.00, 1.13)	1	1.10 (1.01, 1.20)	1.12 (1.02, 1.22)	

Supplementary Table 4. Univariable and multivariable adjusted odds ratios for cause-specific mortality according to BMI on a categorical scale. Model I unadjusted, model II adjusted for age, sex and ethnicity, model III adjusted for age, sex, ethnicity, heart rate, mean blood pressure, APACHE score, GCS, primary reason for admission (cardiovascular disease, respiratory disease, digestive disease, genitourinary disease, neurological disease, endocrine disease, trauma, other infectious disease), preadmission comorbidities (coronary artery disease, stroke/transient ischemic attacks, diabetes mellitus, hypertension, congestive heart failure, peripheral artery disease, chronic obstructive pulmonary disease, renal dysfunction), mechanical ventilation, dialysis, vasoactive drugs, admission source, geographic location and discharge year.

BMI category	Underweight <18.5 kg/m^2	normal weight 18.5-24.9 kg/m^2	Overweight 25-29.9 kg/m^2	class I obesity 30-34.9 kg/m^2	class II obesity 35-39.9 kg/m^2	class III obesity ≥40 kg/m^2
	OR (95% CI)	OR (95% CI)	OR (95% CI)	Reference	OR (95% CI)	OR (95% CI)
**Cardiovascular mortality**						
**Overall population**						
Model I	1.22 (1.07, 1.39)	1.02 (0.94, 1.10)	1.03 (0.95, 1.11)	1	1.03 (0.92, 1.15)	0.97 (0.87, 1.09)
Model II	1.14 (1.00, 1.30)	0.95 (0.87, 1.03)	0.97 (0.90, 1.05)	1	1.09 (0.98, 1.22)	1.12 (1.00, 1.25)
Model III	1.73 (1.46, 2.05)	1.23 (1.12, 1.36)	1.03 (0.94, 1.13)	1	1.09 (0.95, 1.23)	1.24 (1.08, 1.43)
**Men**						
Model I	1.26 (1.03, 1.53)	1.04 (0.93, 1.15)	1.06 (0.96, 1.18)	1	1.01 (0.87, 1.17)	1.10 (0.93, 1.29)
Model II	1.22 (1.00, 1.49)	0.96 (0.86, 1.07)	1.00 (0.90, 1.11)	1	1.07 (0.92, 1.24)	1.26 (1.07, 1.48)
Model III	2.36 (1.83, 3.05)	1.36 (1.19, 1.54)	1.14 (1.00, 1.29)	1	1.06 (0.89, 1.27)	1.51 (1.23, 1.84)
**Women**						
Model I	1.18 (0.98, 1.42)	0.99 (0.88, 1.12)	0.98 (0.87, 1.11)	1	1.04 (0.89, 1.22)	0.88 (0.75, 1.03)
Model II	1.08 (0.90, 1.30)	0.93 (0.83, 1.05)	0.94 (0.83, 1.06)	1	1.11 (0.95, 1.29)	1.01 (0.86, 1.19)
Model III	1.41 (1.12, 1.77)	1.07 (0.93, 1.24)	0.89 (0.77, 1.03)	1	1.08 (0.89, 1.30)	1.03 (0.85, 1.24)
**Infectious mortality**						
**Overall population**						
Model I	2.68 (2.36, 3.05)	1.57 (1.43, 1.73)	1.11 (1.01, 1.23)	1	0.98 (0.86, 1.13)	1.09 (0.95, 1.25)
Model II	2.48 (2.18, 2.83)	1.44 (1.31, 1.59)	1.04 (0.95, 1.15)	1	1.05 (0.91, 1.20)	1.27 (1.11, 1.46)
Model III	1.67 (1.45, 1.93)	1.23 (1.11, 1.36)	1.06 (0.95, 1.18)	1	0.92 (0.79, 1.07)	0.94 (0.81, 1.09)
**Men**						
Model I	3.44 (2.87, 4.12)	1.67 (1.47, 1.90)	1.13 (0.99, 1.29)	1	0.99 (0.81, 1.20)	1.11 (0.90, 1.37)

Model II	3.35 (2.79, 4.02)	1.52 (1.33, 1.73)	1.04 (0.91, 1.19)	1	1.06 (0.87, 1.29)	1.32 (1.07, 1.63)
Model III	1.92 (1.56, 2.35)	1.19 (1.03, 1.37)	1.04 (0.90, 1.21)	1	0.92 (0.74, 1.14)	0.92 (0.74, 1.16)
**Women**						
Model I	2.11 (1.76, 2.55)	1.46 (1.27, 1.67)	1.10 (0.95, 1.28)	1	0.96 (0.79, 1.16)	1.04 (0.86, 1.25)
Model II	1.91 (1.58, 2.30)	1.35 (1.17, 1.55)	1.05 (0.91, 1.22)	1	1.02 (0.84, 1.24)	1.20 (1.00, 1.45)
Model III	1.48 (1.20, 1.82)	1.28 (1.10, 1.49)	1.07 (0.91, 1.26)	1	0.92 (0.74, 1.14)	0.95 (0.78, 1.16)
**Other-cause mortality**						
**Overall population**						
Model I	2.13 (1.87, 2.44)	1.48 (1.35, 1.62)	1.12 (1.02, 1.23)	1	1.19 (1.05, 1.34)	1.18 (1.04, 1.35)
Model II	2.05 (1.80, 2.35)	1.41 (1.29, 1.55)	1.08 (0.98, 1.18)	1	1.24 (1.10, 1.41)	1.32 (1.16, 1.50)
Model III	1.38 (1.20, 1.59)	1.11 (1.01, 1.22)	1.01 (0.91, 1.11)	1	1.20 (1.06, 1.37)	1.24 (1.08, 1.41)
**Men**						
Model I	1.90 (1.56, 2.32)	1.49 (1.32, 1.67)	1.05 (0.93, 1.19)	1	1.13 (0.96, 1.34)	1.17 (0.97, 1.41)
Model II	1.90 (1.56, 2.32)	1.43 (1.27, 1.61)	1.01 (0.89, 1.14)	1	1.18 (0.99, 1.39)	1.28 (1.06, 1.55)
Model III	1.30 (1.10, 1.59)	1.08 (0.95, 1.22)	0.93 (0.82, 1.06)	1	1.17 (0.98, 1.41)	1.16 (0.95, 1.42)
**Women**						
Model I	2.37 (1.97, 2.84)	1.49 (1.29, 1.71)	1.23 (1.07, 1.43)	1	1.26 (1.05, 1.51)	1.22 (1.02, 1.46)
Model II	2.19 (1.82, 2.63)	1.40 (1.22, 1.61)	1.19 (1.03, 1.38)	1	1.33 (1.11, 1.59)	1.38 (1.15, 1.65)
Model III	1.61 (1.33, 1.96)	1.16 (1.00, 1.34)	1.12 (0.96, 1.30)	1	1.25 (1.03, 1.52)	1.33 (1.10, 1.61)

Supplementary Table 5. Univariable and multivariable adjusted odds ratios for all-cause and cause-specific mortality according to BMI on a categorical scale after exclusion of deaths within 48 hours of admission. Model I unadjusted, model II adjusted for age, sex and ethnicity, model III adjusted for age, sex, ethnicity, heart rate, mean blood pressure, APACHE score, GCS, primary reason for admission (cardiovascular disease, respiratory disease, digestive disease, genitourinary disease, neurological disease, endocrine disease, trauma, other infectious disease), preadmission comorbidities (coronary artery disease, stroke/transient ischemic attacks, diabetes mellitus, hypertension, congestive heart failure, peripheral artery disease, chronic obstructive pulmonary disease, renal dysfunction), mechanical ventilation, dialysis, vasoactive drugs, admission source, geographic location and discharge year.

BMI category	Underweight <18.5 kg/m^2	normal weight 18.5-24.9 kg/m^2	Overweight 25-29.9 kg/m^2	class I obesity 30-34.9 kg/m^2	class II obesity 35-39.9 kg/m^2	class III obesity ≥40 kg/m^2
	OR (95% CI)	OR (95% CI)	OR (95% CI)	Reference	OR (95% CI)	OR (95% CI)
**All-cause mortality**						
**Overall population**						
Model I	1.85 (1.67, 2.03)	1.26 (1.18, 1.35)	1.06 (0.99, 1.13)	1	1.05 (0.96, 1.14)	1.09 (0.99, 1.19)
Model II	1.76 (1.60, 1.94)	1.19 (1.12, 1.27)	1.00 (0.94, 1.07)	1	1.11 (1.01, 1.21)	1.24 (1.14, 1.36)
Model III	1.49 (1.34, 1.65)	1.15 (1.07, 1.23)	1.02 (0.95, 1.09)	1	1.06 (0.96, 1.16)	1.12 (1.01, 1.23)
**Men**						
Model I	2.01 (1.75, 2.32)	1.32 (1.22, 1.44)	1.05 (0.96, 1.14)	1	1.05 (0.93, 1.19)	1.13 (0.99, 1.29)
Model II	1.99 (1.73, 2.30)	1.24 (1.14, 1.35)	0.98 (0.90, 1.07)	1	1.11 (0.99, 1.26)	1.30 (1.13, 1.48)
Model III	1.62 (1.39, 1.89)	1.18 (1.08, 1.30)	1.02 (0.93, 1.12)	1	1.07 (0.94, 1.22)	1.14 (1.00, 1.32)
**Women**						
Model I	1.71 (1.49, 1.96)	1.19 (1.08, 1.31)	1.07 (0.97, 1.18)	1	1.04 (0.91, 1.18)	1.05 (0.92, 1.18)
Model II	1.59 (1.38, 1.82)	1.13 (1.02, 1.24)	1.03 (0.93, 1.14)	1	1.10 (0.97, 1.25)	1.19 (1.05, 1.35)

Model III	1.38 (1.19, 1.59)	1.10 (0.99, 1.22)	1.02 (0.92, 1.14)	1	1.03 (0.90, 1.18)	1.08 (0.94, 1.23)
**Cardiovascular mortality**						
**Overall population**						
Model I	1.03 (0.86, 1.23)	0.96 (0.87, 1.06)	1.00 (0.90, 1.11)	1	1.04 (0.91, 1.19)	1.02 (0.88, 1.17)
Model II	0.97 (0.81, 1.16)	0.90 (0.81, 1.03)	0.95 (0.86, 1.05)	1	1.10 (0.96, 1.26)	1.16 (1.01, 1.33)
Model III	1.45 (1.18, 1.79)	1.18 (1.05, 1.32)	1.03 (0.92, 1.15)	1	1.09 (0.93, 1.26)	1.25 (1.06, 1.46)
**Men**						
Model I	1.03 (0.78, 1.35)	0.98 (0.85, 1.12)	1.01 (0.89, 1.15)	1	1.01 (0.84, 1.22)	1.19 (0.97, 1.45)
Model II	1.00 (0.76, 1.31)	0.91 (0.79, 1.04)	0.95 (0.83, 1.09)	1	1.07 (0.88, 1.29)	1.35 (1.11, 1.65)
Model III	1.88 (1.37, 2.58)	1.29 (1.11, 1.50)	1.11 (0.95, 1.28)	1	1.06 (0.86, 1.30)	1.52 (1.21, 1.91)
**Women**						
Model I	1.03 (0.80, 1.31)	0.94 (0.81, 1.10)	0.98 (0.84, 1.15)	1	1.07 (0.88, 1.30)	0.89 (0.73, 1.09)
Model II	1.03 (0.81, 1.32)	0.95 (0.81, 1.10)	0.99 (0.84, 1.15)	1	1.06 (0.88, 1.29)	0.89 (0.73, 1.09)
Model III	1.38 (1.05, 1.82)	1.10 (0.93, 1.30)	0.95 (0.80, 1.13)	1	1.02 (0.82, 1.26)	0.94 (0.76, 1.17)
**Infectious mortality**						
**Overall population**						
Model I	2.53 (2.15, 2.97)	1.51 (1.34, 1.69)	1.08 (0.95, 1.22)	1	0.93 (0.79, 1.11)	1.09 (0.92, 1.28)
Model II	2.37 (2.01, 2.78)	1.40 (1.24, 1.57)	1.01 (0.90, 1.14)	1	1.00 (0.84, 1.18)	1.27 (1.07, 1.50)
Model III	1.52 (1.27, 1.80)	1.19 (1.05, 1.34)	1.03 (0.91, 1.17)	1	0.88 (0.73, 1.05)	0.93 (0.78, 1.11)
**Men**						
Model I	3.45 (2.76, 4.30)	1.65 (1.41, 1.93)	1.09 (0.92, 1.28)	1	1.00 (0.79, 1.27)	1.09 (0.84, 1.41)
Model II	3.32 (2.65, 4.15)	1.49 (1.27, 1.74)	1.00 (0.85, 1.18)	1	1.07 (0.85, 1.36)	1.30 (1.00, 1.68)
Model III	1.79 (1.41, 2.28)	1.16 (0.98, 1.37)	1.01 (0.85, 1.20)	1	0.93 (0.72, 1.19)	0.90 (0.69, 1.19)
**Women**						
Model I	1.86 (1.47, 2.36)	1.35 (1.14, 1.60)	1.07 (0.89, 1.28)	1	0.86 (0.67, 1.10)	1.05 (0.83, 1.31)
Model II	1.71 (1.34, 2.17)	1.27 (1.07, 1.50)	1.02 (0.85, 1.23)	1	0.91 (0.71, 1.17)	1.20 (0.96, 1.51)
Model III	1.35(1.05, 1.64)	1.20 (1.00, 1.44)	1.05 (0.87, 1.27)	1	0.82 (0.63, 1.06)	0.93 (0.73, 1.18)
**Other-cause mortality**						
**Overall population**						
Model I	2.17 (1.85, 2.55)	1.41 (1.26, 1.57)	1.11 (0.99, 1.24)	1	1.15 (0.99, 1.34)	1.17 (1.00, 1.37)
Model II	2.11 (1.79, 2.47)	1.35 (1.21, 1.51)	1.06 (0.95, 1.19)	1	1.20 (1.03, 1.40)	1.30 (1.11, 1.52)
Model III	1.39 (1.18, 1.65)	1.08 (0.96, 1.21)	1.01 (0.89, 1.13)	1	1.16 (0.99, 1.35)	1.19 (1.02, 1.40)
**Men**						
Model I	1.95 (1.53, 2.48)	1.47 (1.27, 1.69)	1.05 (0.90, 1.22)	1	1.15 (0.93, 1.41)	1.08 (0.86, 1.37)
Model II	1.96 (1.54, 2.50)	1.41 (1.22, 1.63)	1.01 (0.87, 1.17)	1	1.19 (0.97, 1.46)	1.19 (0.94, 1.50)
Model III	1.30 (1.08, 1.54)	1.08 (0.93, 1.26)	0.94 (0.81, 1.10)	1	1.18 (0.95, 1.46)	1.06 (0.83, 1.36)
**Women**						
Model I	2.37 (1.91, 2.95)	1.35 (1.14, 1.59)	1.19 (1.00, 1.41)	1	1.16 (0.93, 1.45)	1.25 (1.01, 1.54)
Model II	2.21 (1.78, 2.76)	1.28 (1.08, 1.52)	1.15 (0.96, 1.37)	1	1.22 (0.98, 1.52)	1.40 (1.13, 1.73)
Model III	1.60 (1.27, 2.01)	1.09 (0.92, 1.30)	1.09 (0.91, 1.31)	1	1.14 (0.90, 1.43)	1.31 (1.05, 1.63)

Supplementary Table 6. Univariable and multivariable adjusted odds ratios for all-cause and cause-specific mortality according to BMI on a categorical scale with complete-case analysis. Model I unadjusted, model II adjusted for age, sex and ethnicity, model III adjusted for age, sex, ethnicity, heart rate, mean blood pressure, APACHE score, GCS, primary reason for admission (cardiovascular disease, respiratory disease, digestive disease, genitourinary disease, neurological disease, endocrine

disease, trauma, other infectious disease), preadmission comorbidities (coronary artery disease, stroke/transient ischemic attacks, diabetes mellitus, hypertension, congestive heart failure, peripheral artery disease, chronic obstructive pulmonary disease, renal dysfunction), mechanical ventilation, dialysis, vasoactive drugs, admission source, geographic location and discharge year.

BMI category	Underweight <18.5 kg/m²	normal weight 18.5-24.9 kg/m²	Overweight 25-29.9 kg/m²	class I obesity 30-34.9 kg/m²	class II obesity 35-39.9 kg/m²	class III obesity ≥40 kg/m²
	OR (95% CI)	OR (95% CI)	OR (95% CI)	Reference	OR (95% CI)	OR (95% CI)
**All-cause mortality**						
**Overall population**						
Model I	2.03 (1.86, 2.21)	1.38 (1.30, 1.46)	1.12 (1.06, 1.19)	1	1.10 (1.02, 1.19)	1.08 (1.00, 1.17)
Model II	1.92 (1.76, 2.10)	1.29 (1.22, 1.37)	1.06 (1.01, 1.13)	1	1.17 (1.08, 1.27)	1.24 (1.14, 1.35)
Model III	1.62 (1.46, 1.79)	1.24 (1.16, 1.32)	1.07 (1.01, 1.15)	1	1.11 (1.01, 1.21)	1.13 (1.03, 1.24)
**Men**						
Model I	2.13 (1.88, 2.42)	1.44 (1.33, 1.55)	1.13 (1.05, 1.22)	1	1.10 (0.99, 1.23)	1.12 (0.99, 1.27)
Model II	2.12 (1.86, 2.41)	1.35 (1.25, 1.45)	1.06 (0.98, 1.15)	1	1.16 (1.04, 1.30)	1.29 (1.14, 1.45)
Model III	1.68 (1.45, 1.95)	1.27 (1.17, 1.39)	1.10 (1.01, 1.20)	1	1.11 (0.98, 1.25)	1.16 (1.01, 1.33)
**Women**						
Model I	1.93 (1.71, 2.17)	1.31 (1.21, 1.43)	1.11 (1.02, 1.21)	1	1.10 (0.98, 1.23)	1.04 (0.93, 1.16)
Model II	1.76 (1.56, 1.99)	1.23 (1.13, 1.34)	1.06 (0.97, 1.16)	1	1.17 (1.04, 1.31)	1.19 (1.07, 1.34)
Model III	1.55 (1.35, 1.77)	1.19 (1.08, 1.31)	1.04 (0.94, 1.15)	1	1.09 (0.96, 1.24)	1.10 (0.97, 1.25)
**Cardiovascular mortality**						
**Overall population**						
Model I	1.22 (1.06, 1.42)	1.06 (0.97, 1.15)	1.05 (0.96, 1.15)	1	1.04 (0.92, 1.17)	0.96 (0.85, 1.09)
Model II	1.14 (0.98, 1.33)	0.98 (0.90, 1.08)	0.99 (0.91, 1.08)	1	1.10 (0.98, 1.24)	1.10 (0.97, 1.25)
Model III	1.68 (1.39, 2.03)	1.29 (1.16, 1.44)	1.07 (0.96, 1.19)	1	1.07 (0.93, 1.24)	1.21 (1.04, 1.42)
**Men**						
Model I	1.27 (1.02, 1.58)	1.09 (0.97, 1.23)	1.10 (0.98, 1.24)	1	1.04 (0.88, 1.22)	1.07 (0.89, 1.29)
Model II	1.24 (0.99, 1.54)	1.02 (0.90, 1.14)	1.04 (0.92, 1.17)	1	1.09 (0.92, 1.29)	1.22 (1.02, 1.47)
Model III	2.31 (1.73, 3.10)	1.43 (1.24, 1.65)	1.22 (1.06, 1.41)	1	1.07 (0.88, 1.31)	1.44 (1.15, 1.81)
**Women**						
Model I	1.17 (0.96, 1.44)	1.02 (0.89, 1.16)	0.98 (0.86, 1.12)	1	1.04 (0.87, 1.23)	0.87 (0.73, 1.04)
Model II	1.07 (0.87, 1.31)	0.95 (0.83, 1.09)	0.94 (0.82, 1.07)	1	1.10 (0.93, 1.30)	1.00 (0.84, 1.19)
Model III	1.35 (1.05, 1.73)	1.07 (0.92, 1.25)	0.87 (0.75, 1.02)	1	1.04 (0.85, 1.28)	1.07 (0.87, 1.31)
**Infectious mortality**						
**Overall population**						
Model I	2.65 (2.30, 3.05)	1.59 (1.44, 1.76)	1.15 (1.03, 1.28)	1	1.02 (0.88, 1.18)	1.08 (0.93, 1.26)
Model II	2.45 (2.12, 2.83)	1.46 (1.32, 1.62)	1.08 (0.97, 1.20)	1	1.09 (0.94, 1.26)	1.26 (1.08, 1.47)
Model III	1.61 (1.37, 1.89)	1.24 (1.11, 1.39)	1.08 (0.96, 1.22)	1	0.94 (0.80, 1.10)	0.92 (0.78, 1.08)
**Men**						
Model I	3.30 (2.70, 4.02)	1.70 (1.48, 1.95)	1.15 (1.00, 1.33)	1	1.03 (0.84, 1.27)	1.11 (0.89, 1.40)
Model II	3.21 (2.62, 3.93)	1.54 (1.34, 1.77)	1.06 (0.92, 1.23)	1	1.11 (0.90, 1.36)	1.32 (1.05, 1.66)
Model III	1.75 (1.39, 2.19)	1.20 (1.03, 1.40)	1.05 (0.90, 1.24)	1	0.94 (0.75, 1.18)	0.91 (0.71, 1.17)
**Women**						
Model I	2.15 (1.75, 2.63)	1.47 (1.26, 1.71)	1.15 (0.98, 1.35)	1	0.99 (0.81, 1.23)	1.03 (0.84, 1.26)

Model II	1.93 (1.58, 2.37)	1.36 (1.17, 1.58)	1.10 (0.94, 1.29)	1	1.06 (0.86, 1.31)	1.19 (0.97, 1.45)
Model III	1.49 (1.19, 1.87)	1.28 (1.08, 1.51)	1.11 (0.94, 1.33)	1	0.93 (0.74, 1.17)	0.92 (0.74, 1.15)
**Other-cause mortality**						
**Overall population**						
Model I	2.31 (1.99, 2.67)	1.57 (1.42, 1.74)	1.18 (1.06, 1.31)	1	1.26 (1.10, 1.45)	1.25 (1.08, 1.44)
Model II	2.21 (1.91, 2.57)	1.50 (1.36, 1.66)	1.13 (1.02, 1.26)	1	1.32 (1.15, 1.51)	1.38 (1.19, 1.59)
Model III	1.46 (1.25, 1.71)	1.16 (1.05, 1.30)	1.06 (0.95, 1.19)	1	1.28 (1.10, 1.48)	1.27 (1.09, 1.48)
**Men**						
Model I	1.97 (1.57, 2.46)	1.60 (1.40, 1.83)	1.13 (0.98, 1.30)	1	1.24 (1.03, 1.50)	1.18 (0.96, 1.46)
Model II	1.98 (1.58, 2.48)	1.54 (1.35, 1.77)	1.09 (0.95, 1.25)	1	1.28 (1.06, 1.55)	1.29 (1.04, 1.59)
Model III	1.30 (1.03, 1.64)	1.23 (1.07, 1.42)	1.03 (0.89, 1.19)	1	1.32 (0.99, 1.61)	1.22 (0.97, 1.52)
**Women**						
Model I	2.58 (2.12, 3.15)	1.54 (1.32, 1.80)	1.25 (1.06, 1.47)	1	1.29 (1.05, 1.58)	1.30 (1.07, 1.58)
Model II	2.38 (1.95, 2.91)	1.45 (1.24, 1.70)	1.20 (1.02, 1.42)	1	1.36 (1.11, 1.66)	1.47 (1.21, 1.79)
Model III	1.75 (1.41, 2.16)	1.20 (1.02, 1.41)	1.14 (0.96, 1.35)	1	1.28 (1.03, 1.58)	1.40 (1.14, 1.72)
